# Supplementary material for: Protocol on a multicentre statistical and economic modelling study of risk-based stratified and personalised screening for diabetes and its complications in India (SMART India)
Source: BMJ Open. 2020 Dec 12;10(12):e039657. doi: 10.1136/bmjopen-2020-039657 (PMC7735124; doi:10.1136/bmjopen-2020-039657)
Supplement: Supplementary data [file bmjopen-2020-039657supp001.pdf]

INFORMED CONSENT FORM
FOR PARTICIPATION IN THE SMART INDIA STUDY

India has the second largest number of people with diabetes in the world and the number is increasing every year. It is well known that people with diabetes are at a higher risk of getting eye problems, heart attack/ stroke, or kidney disease. Some people may have altered blood sugar levels before they actually develop diabetes. This is an All India study which is being done to find out the burden of pre-diabetes and diabetes and the complications due to diabetes, especially the eye complication of diabetes called retinopathy. For this purpose you will be asked some questions which will be recorded in a questionnaire. Blood pressure and a few anthropometric measurements will be taken. All people will then have a finger prick blood test done and photo of the back of the eye (retina) taken using a simple retinal camera. Some additional blood tests and urine test will be done for a subset of people. It is possible that this study could determine that you have diabetes and / or its associated disorders. If so, you will benefit from this information as you can seek early treatment for these disorders. The information you provide in the questionnaire, results of your blood tests and retinal photography will be kept confidential.

**Patient identification number for this study**	
**Title of the project**	**SMART INDIA study**(**S**tatistical **M**odelling and **R**isk **A**ssessment of **T**ype 2 diabetes complications in India)
**Name of Principal Investigator (s)**	

The contents of the patient information sheet that has been provided have been read carefully by me/explained in detail to me, in a language that I comprehend, and I have fully understood the contents.
I confirm that I have had the opportunity to ask questions. The nature and purpose of the study and its potential risks / benefits and expected duration of the study, and other relevant details have been explained to me in detail. I understand that my participation in this study is voluntary and that I am free to withdraw at any time, without giving any reason.
I understand that the information collected about me from participation in this study and sections of any of the results may be looked at by responsible individuals involved in this research project either in India or outside India. Anonymised data and retinal images may be shared with other researchers.
I agree to take part in the above study.

-- Date:
(Signature/Left Thumb impression of participant)
Place: Plac

Name of the Participant: _ _ _ _ _ _ _ _ _ _ _ _ _ _ _ _ _ _

Son/Daughter/spouse of: _ _ _ _ _ _ _ _ _ _ _ _ _ _ _ _ _ _

Complete postal address: _ _ _ _ _ _ _ _ _ _ _ _ _ _ _ _ _ _

_ _ _
_ _ _

1) Witness

-------------------------------------- Date:
(Signature)

Name

Address:

Sivaprasad S, *et al. BMJ Open* 2020; 10:e039657. doi: 10.1136/bmjopen-2020-039657
