# Supplementary material for: Protocol on a multicentre statistical and economic modelling study of risk-based stratified and personalised screening for diabetes and its complications in India (SMART India)
Source: BMJ Open. 2020 Dec 12;10(12):e039657. doi: 10.1136/bmjopen-2020-039657 (PMC7735124; doi:10.1136/bmjopen-2020-039657)
Supplement: Supplementary data [file bmjopen-2020-039657supp002.pdf]

SMART India	Participant ID:							Participant Initials		
	Date of Consent:							Year of birth:		

SMART India study
Questionnaire

* All questionnaires must be interviewer administered

S.No	Check List	YES	NO
1	Household details		
2	Demographic data and Anthropometric measurements (Main survey)		
3	Diabetes Information		
4	EQ5D questionnaire		
5	Vision Quality of Life questionnaire (VisQoL)		
6	Cost data/Expenses form		
7	Fundus Image		

Person administering the questionnaire	
**Signature**	
**Name**	
**Participant who is administered**	
**Signature**	
**Name**	

Sivaprasad S, *et al. BMJ Open* 2020; 10:e039657. doi: 10.1136/bmjopen-2020-039657

SMART India	Participant ID:							Participant Initials		
	Date of Consent:							Year of birth:		

PART 1 – House hold Details- House Survey Record

1	Centre		
2	Region Type	1	Urban
		2	Rural
		3	Special
3	Address		
4	Phone / Mobile Number:		
5	City		
6	Pin		
7	Household Status	1	No one available in this household
		2	Household not willing to participate
		3	Available

*If **1** or **2** skip question No.**8***

8	If available, number of people in house above 40 years	
9	Enter details of people in the house hold	

SMART India	Participant ID:							Participant Initials		
	Date of Consent:							Year of birth:		

**9.1. Person-1**			
a	Participation	1	Willing to take part
		2	Type 1 diabetic - exclusion
		3	Gestational diabetes - exclusion
		4	Other exclusion
	If 4, Reason		

*If **3** skip "**b**", if **1 or 2** skip "**c and d**"*

b	Gender	1	Male
		2	Female
		3	Other (do not want to disclose, transgender, etc.)

c	Name of the person 1<br>*Example: **A**jith **K**umar*	

d	Initials of the person 1<br>*Example: **AK***	

**9.2 Person-2**			
a	Participation	1	Willing to take part
		2	Type 1 diabetic - exclusion
		3	Gestational diabetes - exclusion
		4	Other exclusion
	If 4, Reason		

*If **3** skip "**b**", if **1 or 2** skip "**c and d**"*

b	Gender	1	Male
		2	Female
		3	Other (do not want to disclose, transgender, etc.)

c	Name of the person 2	

d	Initials of the person 2	

Sivaprasad S, *et al. BMJ Open* 2020; 10:e039657. doi: 10.1136/bmjopen-2020-039657

**SMART India**	**Participant ID:**							**Participant Initials**			
	**Date of Consent:**							**Year of birth:**			

**9.3. Person-3**			
a	Participation	1	Willing to take part
		2	Type 1 diabetic - exclusion
		3	Gestational diabetes - exclusion
		4	Other exclusion
	If 4, Reason		

*If **3** skip "**b**", if **1 or 2** skip "**c and d**"*

b	Gender	1	Male
		2	Female
		3	Other (do not want to disclose, transgender, etc.)

c	Name of the person 3	

d	Initials of the person 3	

**9.4. Person-4**			
a	Participation	1	Willing to take part
		2	Type 1 diabetic - exclusion
		3	Gestational diabetes - exclusion
		4	Other exclusion
	If 4, Reason		

*If **3** skip "**b**", if **1 or 2** skip "**c** and **d**"*

b	Gender	1	Male
		2	Female
		3	Other (do not want to disclose, transgender, etc.)

c	Name of the person 4	

d	Initials of the person 4	

Sivaprasad S, *et al. BMJ Open* 2020; 10:e039657. doi: 10.1136/bmjopen-2020-039657

SMART India	Participant ID:							Participant Initials				
	Date of Consent:							Year of birth:				

9.5. Person -5			
a	Participation	1	Willing to take part
		2	Type 1 diabetic - exclusion
		3	Gestational diabetes - exclusion
		4	Other exclusion
	If 4, Reason		

*If **3** skip "**b**", if **1 or 2** skip "**c** and **d**"*

b	Gender	1	Male
		2	Female
		3	Other (do not want to disclose, transgender, etc.)

c	Name of the person 5	

d	Initials of the person 5	

9.6. Person - 6			
a	Participation	1	Willing to take part
		2	Type 1 diabetic - exclusion
		3	Gestational diabetes - exclusion
		4	Other exclusion
	If 4, Reason		

*If **3** skip "**b**", if **1 or 2** skip "**c** and **d**"*

b	Gender	1	Male
		2	Female
		3	Other (do not want to disclose, transgender, etc.)

c	Name of the person 6	

d	Initials of the person 6	

SMART India	Participant ID:							Participant Initials			
	Date of Consent:							Year of birth:			

PART 2 – Demographic data and Anthropometric measurements

Instructions:

READ CATEGORIES for all questions. CIRCLE ONE

1	Participant ID:		
2	Date of Consent:		_ _ / _ _ / _ _ _ _
3	Year of Birth:		

Note: *Choose between 1920 to 1978*

4	Gender:	1	Male
		2	Female
		3	Other (do not want to disclose, transgender, etc.)

5	Highest level of Education: (Select Education Level)	1	None
		2	Primary
		3	Secondary
		4	Graduate
		5	Postgraduate or higher
		6	Not classified

6	Occupation: (select occupation)	1	Not working due to health reasons
		2	Not working due to vision reasons
		3	Housewife
		4	Unemployed
		5	Retired
		6	Unskilled worker
		7	Skilled worker
		8	Professional
		9	Self Employed

Sivaprasad S, *et al. BMJ Open* 2020; 10:e039657. doi: 10.1136/bmjopen-2020-039657

**SMART India**	**Participant ID:**							**Participant Initials**		
	**Date of Consent:**							**Year of birth:**		

7	Average Monthly Individual Income (Rs.)	1	Do not want to disclose
		2	
			Enter valid Income (0-10000000)
8	Smoking Status	1	Non-smoker
		2	Former smoker
		3	Smoker
			If ***1*** *or* ***2*** *Go to* ***9***
8a	No of cigarettes per day:		
			Please enter valid value (1-99)
9	Second hand smoke exposure for one or more hours per week:	1	No
		2	Yes
10	Physical Exercise (Select from list)	1	Sedentary
		2	Mild exercise
		3	Moderate exercise
		4	Vigorous or strenuous exercise
11	Several periods of stress or permanent stress in the last year (select Yes or No)	1	No
		2	Yes
12	In the last year, was there a time when you felt sad, blue or depressed for two weeks or more in a row (*select Yes or No*)	1	No
		2	Yes
13	Diet: (Select all that applies)<br><br>At least one option should be selected.	1	Salty food or snacks one or more times a day
		2	Deep fried foods or snacks or fast foods 3 or more times per week
		3	Eat fruit less than once per day
		4	Eat vegetables less than once per day
		5	Eat meat and / or poultry 2 or more times daily
		6	None of the above

Sivaprasad S, *et al. BMJ Open* 2020; 10:e039657. doi: 10.1136/bmjopen-2020-039657

SMART India	Participant ID:							Participant Initials		
	Date of Consent:							Year of birth:		

14	Diagnosed diabetes Type 2 (Only Type 2 eligible)	1	Don’t know
		2	No
		3	Yes

If “Don’t Know or NO ” Go to ***15***

14a	Duration of diabetes Type 2 since diagnosis. (enter duration in years and 0 – 11 months)	Years:	Months:

14b	Treatment of Diabetes Mellitus:	1	None / Diet controlled
		2	Oral hypoglycaemic agents only
		3	Insulin only
		4	Both insulin and oral hypoglycaemic agent

14c	Complications of diabetes mellitus (Select all that applies)	1	None
		2	Chronic kidney disease
		3	Peripheral neuropathy (diabetic foot)
		4	Diabetic retinopathy

14d	Are you aware that diabetes can cause blindness?	1	No
		2	Yes

15	Cardiovascular disease (Select all that applies)	1	None
		2	Hypertension
		3	Myocardial infarction
		4	Heart failure
		5	Stroke
		6	Transient ischaemic attack

16	Medical History - any other history not covered above	

17	Ocular history (Select all that applies): *At least one option should be selected*	1	None
		2	Cataract present
		3	Cataract surgery done in at least 1 eye
		4	Glaucoma
		5	AMD (age related macular degeneration)

**SMART India**	**Participant ID:**							**Participant Initials**				
	**Date of Consent:**							**Year of birth:**				

17a	Other Ocular History - *any other history not covered before*	

18	Parental history of diabetes	1	Both non-diabetic
		2	Either parents diabetic
		3	Both parents diabetic

19	Parental history of heart attack	1	No
		2	Yes

20	Height (cms)<br>*Enter Valid Height in cms (100-230)*	

21	Weight (kgs)<br>*Enter valid weight in kgs (30-300)*	

22	Waist circumference (cms)<br>*Enter valid value in cms (20-300)*	

23	Hip circumference (cms)<br>*Enter valid value in cms (20-300)*	

24	Systolic Blood pressure (mm Hg)<br>*Enter valid value (30 - 250) and above Diastolic*	

25	Diastolic Blood pressure (mm Hg)<br>*Enter valid value (30 - 250)*	

SMART India	Participant ID:							Participant Initials		
	Date of Consent:							Year of birth:		

Part 3- Diabetes Information

1	Participant ID:		

2	Diabetes:	1	No/Don't know
		2	Yes

3	Random Blood sugar (mg/dl):<br>*Enter valid value (50 - 500)*	

If patient is known diabetic, then whatever the value of RBS, all tests must be carried out.
If diabetes 'No / Unknown – RBS < 110 – (End of Survey)
RBS between 110 and 160 – Answer 'Carry out all tests ?' Yes – No

3a	Carry Out All Tests?	1	No
		2	Yes

4	HbA1c (%):<br>*Enter valid value (4-13)*	

5	Microalbuminuria:	1	No
		2	Yes
		3	Urine sample not available

6	Total Cholesterol – mg/dL<br>*Enter Valid value (100-400)*	
7	HDL Cholesterol – mg/dL<br>*Enter Valid value (20-120)*	
8	Total Triglycerides – mg/dL<br>*Enter Valid value (50-500)*	
9	LDL Cholesterol – mg/dL<br>*Enter Valid value (0-450)*	
10	Total Cholesterol / HDL Ratio<br>*Enter Valid value (1-33.3)*	
11	Non-HDL Cholesterol – mg/dL<br>*Enter Valid value (0-450)*	

Sivaprasad S, *et al. BMJ Open* 2020; 10:e039657. doi: 10.1136/bmjopen-2020-039657

SMART India	Participant ID:							Participant Initials		
	Date of Consent:							Year of birth:		

12	Distance Vision in right eye (with glasses if available) *Select from list*	1	0.0
		2	0.1
		3	0.2
		4	0.3
		5	0.4
		6	0.5
		7	0.6
		8	0.7
		9	0.8
		10	0.9
		11	1.0
		12	1.1
		13	1.2
		14	Worse than or equal to 1.3

13	Distance Vision in left eye (with glasses if available) *Select from list*	1	0.0
		2	0.1
		3	0.2
		4	0.3
		5	0.4
		6	0.5
		7	0.6
		8	0.7
		9	0.8
		10	0.9
		11	1.0
		12	1.1
		13	1.2
		14	Worse than or equal to 1.3

14	Were the fundus photographs taken? *Please enter the Participant ID in fundus system*	1	Yes
		2	Not obtainable
NOTE: If 2 :Please capture the participants front of the eye and upload it in the upload page, if the image is not obtainable			

SMART India	Participant ID:							Participant Initials		
	Date of Consent:							Year of birth:		

PART 4 – Eq5d questionnaire

By placing a tick in one box in each group below, please indicate which statements best describe your own health state **TODAY**

1	Mobility	1	I have no problems in walking about
		2	I have slight problems in walking about
		3	I have moderate problems in walking about
		4	I have severe problems in walking about
		5	I am unable to walk about

2	Self-care	1	I have no problems washing or dressing myself
		2	I have mild problems washing or dressing myself
		3	I have moderate problems washing or dressing myself
		4	I have severe problems washing or dressing myself
		5	I am unable to wash or dress myself

3	Usual Activities (*e.g. work, study, housework, family or leisure activities)*	1	I have no problems with performing my usual activities
		2	I have mild problems with performing my usual activities
		3	I have moderate problems with performing my usual activities
		4	I have severe problems with performing my usual activities
		5	I am unable to perform my usual activities

4	Pain / Discomfort	1	I have no pain or discomfort
		2	I have mild pain or discomfort
		3	I have moderate pain or discomfort
		4	I have severe pain or discomfort
		5	I have extreme pain or discomfort

5	Anxiety / Depression	1	I am not anxious or depressed
		2	I am mildly anxious or depressed
		3	I am moderately anxious or depressed
		4	I am severely anxious or depressed
		5	I am extremely anxious or depressed

6	Vision *(using glasses or contact lenses if needed)*	1	I have no problems seeing
		2	I have slight problems seeing
		3	I have some problems seeing
		4	I have severe problems seeing
		5	I am unable to see

Sivaprasad S, *et al. BMJ Open* 2020; 10:e039657. doi: 10.1136/bmjopen-2020-039657

SMART India	Participant ID:							Participant Initials		
	Date of Consent:							Year of birth:		

7	How good or bad your health state, is imagined in a scale 0 to 100. *The best state you can imagine is written as 100 and the worst state you can imagine is written as 0.*	

Enter value between (0 – 100)

8	Life satisfaction: All things considered, how satisfied are you with your life as a whole these days in 1 to 10 scale? *Please mark on the scale where 1 is dissatisfied* and 10 is satisfied.	

Enter value between(0 – 10)

Part 5 - Vision quality of life questionnaire

1	Does my vision make it likely I will injure myself (i.e., when moving around the house, yard, neighbourhood, or workplace)?	1	It is most unlikely I will injure myself because of my vision
		2	There is a small chance
		3	There is a good chance
		4	It is very likely
		5	Almost certainly my vision will cause me to injure myself

2	Does my vision make it difficult to cope with the demands in my life?<br><br>My vision:	1	Has no effect on my ability to cope with the demands in my life
		2	Does not make it difficult at all to cope with the demands in my life
		3	Makes it a little difficult to cope
		4	Makes it moderately difficult to cope
		5	Makes it very difficult to cope
		6	Makes me unable to cope at all

3	Does my vision affect my ability to have friendships?<br><br>My vision:	1	Makes having friendships easier
		2	Has no effect on my friendships
		3	Makes friendships more difficult
		4	Makes friendships a lot more difficult

SMART India	Participant ID:		Participant Initials	
	Date of Consent:		Year of birth:	

		5	Makes friendships extremely difficult
		6	Makes me unable to have friendships
		7	Not applicable; I have no friendships

4	Do I have difficulty organizing any assistance I may need?	1	I have no difficulty organizing any assistance I may need
		2	I have a little difficulty organizing assistance
		3	I have moderate difficulty organizing assistance
		4	I have a lot of difficulty organizing assistance
		5	I am unable to organize assistance at all
		6	Not applicable; I never need to organize assistance

5	Does my vision make it difficult to fulfil the roles I would like to fulfil in life (e.g., family roles, work roles, community roles)?<br><br>My vision:	1	Has no effect on my ability to fulfil these roles
		2	Does not make it difficult to fulfil these roles
		3	Makes it a little difficult to fulfil these roles
		4	Makes it moderately difficult to fulfil these roles
		5	Makes it very difficult to fulfil these roles
		6	Means I am unable to fulfil these roles

6	Does my vision affect my confidence to join in everyday activities?<br><br>My vision:	1	Makes me more confident to join in everyday activities
		2	Has no effect on my confidence to join in everyday activities
		3	Makes me feel a little less confident
		4	Makes me feel moderately less confident
		5	Makes me feel a lot less confident
		6	Makes me not confident at all

SMART India	Participant ID:							Participant Initials		
	Date of Consent:							Year of birth:		

Part 6 - Expense form

Instructions: Fill the expenses form only for those who are diabetic (if PART 2: 14 = "YES")

1a	Have you seen an eye doctor for diabetic eye disease in the last 3 years?	1	No
		2	Yes

*If 'No' skip all question in expense form (skip **1b to 4**)*

1b	Have you been diagnosed with diabetic eye disease?	1	No
		2	Yes

If 'No' skip to 2a question

1c	Have you received any treatment for diabetic eye disease in the last one year? (*Select all that applies*)	1	No Treatment
		2	Laser (Macular / PRP)
		3	Injection into the Eye (Anti-VEGF / Steroids)
		4	Surgery (Vitrectomy)

At least one option should be selected

1d	How was your vision before treatment?	1	I had no problems seeing
		2	I had slight problems seeing
		3	I had some problems seeing
		4	I had severe problems seeing
		5	I was unable to see

1e	Have you noticed an improvement in your vision following treatment?	1	No change
		2	Improved
		3	Worsened

2a	What were the total costs in last one year for treatment of diabetic eye disease (treatment / consultation / surgery)	Rs.	

Enter valid number (>= 0 and less than 999999)

2b	If you received any treatment including consultations in the last one year for diabetic eye disease, was the treatment	1	Free
		2	Concessional Cost
		3	Paid In Full

3	What were the travel costs for you and your carer (family member) in the last one year to go to the eye doctors, eye hospitals etc. for treatment of diabetic eye disease	Rs.	

Sivaprasad S, et al. BMJ Open 2020; 10:e039657. doi: 10.1136/bmjopen-2020-039657

SMART India	Participant ID:							Participant Initials		
	Date of Consent:							Year of birth:		

Enter valid number (>= 0 and less than 999999)

4	Did you have to take time off work due to diabetic eye disease treatment in the last one year?	1	No
		2	Yes

5a	Do you think you have visual impairment?	1	No
		2	Yes
5b	Does your visual impairment affect your ability to work?	1	No
		2	Yes

6	Did you receive any inpatient treatment for kidney disease in the last one year?	1	No
		2	Yes

7	Did you receive any inpatient treatment for heart condition or stroke in the last one year?	1	No
		2	Yes

8	Did you receive any treatment for diabetic foot disease (Ulcer / Gangrene/ Amputation) in the last one year?	1	No
		2	Yes

9 a	What were the costs in last one year for treatment of diabetes or its complications (heart conditions, kidney problems, feet problems etc) other than diabetic eye disease		
	Break Up	Medications	Rs.
		Investigations	Rs.
		Consultations	Rs.
		Hospitalization	Rs.
		Sum	Rs.
	Or		
	Total	Rs.	

9 b	If you received any treatment in the last one year for diabetes or its complications (heart conditions, kidney problems, feet problems etc), was the treatment…	1	Free
		2	Concessional Cost
		3	Pain In Full

10	What were the travel costs for you and your carer (family member) in the last one year to go to the doctors, hospitals etc for treatment of diabetes or its complications (exclude diabetic eye disease costs)	Rs.	

Enter valid number (>= 0 and less than 999999)

16

SMART India	Participant ID:							Participant Initials		
	Date of Consent:							Year of birth:		

11	Did you have to take time off work due to diabetes or its complications treatment (other than diabetic eye disease) in the last one year?	1	No
		2	Yes

PART 7 - Fundus Image

Instruction:

Please enter the Participant ID in fundus system. Capture Macula centered and Disc centered images and upload minimum 4 images of good quality to the database.

Please capture the participant's front of the eye and upload it in the upload page, **if the image is not obtainable.**

Please write the Fundus cam image ID if unable to transfer the image to database

Image No	Image ID	
	OD	OS
1		
2		
3		
4		
5		
6		
7		
8		
