# Supplementary material for: Protocol on a multicentre statistical and economic modelling study of risk-based stratified and personalised screening for diabetes and its complications in India (SMART India)
Source: BMJ Open. 2020 Dec 12;10(12):e039657. doi: 10.1136/bmjopen-2020-039657 (PMC7735124; doi:10.1136/bmjopen-2020-039657)
Supplement: Author's manuscript [file bmjopen-2020-039657.draft_revisions.pdf]

BMJ Open is committed to open peer review. As part of this commitment we make the peer review history of every article we publish publicly available.

When an article is published we post the peer reviewers' comments and the authors' responses online. We also post the versions of the paper that were used during peer review. These are the versions that the peer review comments apply to.

The versions of the paper that follow are the versions that were submitted during the peer review process. They are not the versions of record or the final published versions. They should not be cited or distributed as the published version of this manuscript.

BMJ Open is an open access journal and the full, final, typeset and author-corrected version of record of the manuscript is available on our site with no access controls, subscription charges or pay-per-view fees (http://bmjopen.bmj.com).

If you have any questions on BMJ Open's open peer review process please email info.bmjopen@bmj.com

BMJ Open

A multi-centre statistical and economic modelling of risk-based stratified and personalised screening for diabetes and its complications in India (SMART India)

Journal:	*BMJ Open*
Manuscript ID	bmjopen-2020-039657
Article Type:	Protocol
Date Submitted by the Author:	21-Apr-2020

Complete List of Authors:	Sivaprasad, Sobha; Moorfields Eye Hospital NHS Foundation Trust, Medical retina<br>Raman, Rajiv; Sankara Nethralaya, Shri Bhagwan Mahavir Vitreoretinal services<br>Rajalakshmi, Ramachandran; Dr Mohans Diabet Special Ctr, Ophthalmology; DR. MOHAN'S DIABETES SPECIALITIES CENTRE AND MADRAS DIABETES RESEARCH FOUNDATION<br>Mohan, V; Madras Diabetes Research Foundation<br>Deepa, Mohan; Madras Diabetes Research Foundation and Dr. Mohan's Diabetes Specialities Centre<br>Das, Taraprasad; 5. Hyderabad Eye Research Foundation, L V Prasad Eye Institute<br>Ramasamy, Kim; Aravind Medical Research Foundation<br>Prevost, A Toby; Imperial College London, Imperial Clinical Trials Unit<br>Wittenberg, Raphael; University of Oxford, Centre for Health Service Economics and Organisation<br>Netuveli, Gopalakrishnan; University of East London, School of Health, Sports and Bioscience<br>Lingam, Gopal; National University Hospital<br>Hanif, Wasim; University Hospital Birmingham<br>Ramakrishnan, Radha; UCL Institute of Ophthalmology<br>Ramu, Jayashree; NIHR Moorfields Biomedical Research Centre, Medical Retina<br>Surya, Janani; Vision Research Foundation, Sankara Nethralaya<br>Conroy, Dolores; UCL Institute of Ophthalmology

Keywords:	Diabetic retinopathy < DIABETES & ENDOCRINOLOGY, General diabetes < DIABETES & ENDOCRINOLOGY, HEALTH ECONOMICS, Medical retina < OPHTHALMOLOGY, Epidemiology < TROPICAL MEDICINE, PUBLIC HEALTH

SCHOLARONE™ Manuscripts

I, the Submitting Author has the right to grant and does grant on behalf of all authors of the Work (as defined in the below author licence), an exclusive licence and/or a non-exclusive licence for contributions from authors who are: i) UK Crown employees; ii) where BMJ has agreed a CC-BY licence shall apply, and/or iii) in accordance with the terms applicable for US Federal Government officers or employees acting as part of their official duties; on a worldwide, perpetual, irrevocable, royalty-free basis to BMJ Publishing Group Ltd ("BMJ") its licensees and where the relevant Journal is co-owned by BMJ to the co-owners of the Journal, to publish the Work in this journal and any other BMJ products and to exploit all rights, as set out in our licence.

The Submitting Author accepts and understands that any supply made under these terms is made by BMJ to the Submitting Author unless you are acting as an employee on behalf of your employer or a postgraduate student of an affiliated institution which is paying any applicable article publishing charge ("APC") for Open Access articles. Where the Submitting Author wishes to make the Work available on an Open Access basis (and intends to pay the relevant APC), the terms of reuse of such Open Access shall be governed by a Creative Commons licence – details of these licences and which Creative Commons licence will apply to this Work are set out in our licence referred to above.

Other than as permitted in any relevant BMJ Author's Self Archiving Policies, I confirm this Work has not been accepted for publication elsewhere, is not being considered for publication elsewhere and does not duplicate material already published. I confirm all authors consent to publication of this Work and authorise the granting of this licence.

For peer review only

A multi-centre statistical and economic modelling of risk-based stratified and personalised screening for diabetes and its complications in India (SMART India)

Sobha Sivaprasad[1, 2], Rajiv Raman[3], Ramachandran Rajalakshmi[4], Viswanathan Mohan[4], Deepa Mohan[4], Taraprasad Das[5], Kim Ramasamy [6], Toby Prevost[7], Raphael Wittenberg[8], Gopal Netuveli,[9] Gopal Lingam[10], Wasim Hanif[11], Radha Ramakrishnan[2], Jayashree Ramu[1], Janani Surya[3] and Dolores Conroy[2] for the SMART India Study Collaborators

1. NIHR Biomedical Research Centre, Moorfields Eye Hospital NHS Foundation Trust, London, UK
2. UCL Institute of Ophthalmology, London, UK
3. Vision Research Foundation, Sankara Nethralaya, Chennai, India
4. Madras Diabetes Research Foundation and Dr Mohan's Diabetes Specialities Centre, Chennai, India
5. Hyderabad Eye Research Foundation, L V Prasad Eye Institute, Hyderabad, India
6. Aravind Medical Research Foundation, Madurai, India
7. Imperial College London, UK
8. University of Oxford, UK
9. University of East London, UK
10. National University Hospital, Singapore
11. University Hospital, Birmingham, UK

Word count: 4450

Tables: 2

Figures: 2

References: 23

Abstract: 290

Corresponding author

Professor Sobha Sivaprasad
NIHR Moorfields Biomedical Research Centre
Moorfields Eye Hospital NHS Foundation Trust,
162 City Road,
London EC1V 2PD, UK
Tel: 020 7253 3411
Email: senswathi@aol.com

Keywords: Diabetes, glycated haemoglobin, diabetic retinopathy, diabetes complications, India

For peer review only

Abstract

INTRODUCTION:

The aim of this study is to develop practical and affordable models to (a) diagnose people with diabetes and pre-diabetes and (b) identify those at risk of diabetes complications so that these models can be applied to the population in low and middle-income countries (LMIC) where laboratory tests are unaffordable.

METHODS AND ANALYSIS:

This statistical and economic modelling study will be done on a prospectively recruited cohort of at least 48,000 participants aged 40 years or above through community screening across 20 pre-defined regions in India. Each participant will be tested for capillary random blood glucose (RBG) and complete a detailed health related questionnaire. People with known diabetes and all participants with pre-defined levels of RBG will undergo further tests including point-of-care (POC) glycated haemoglobin (HbA1c), POC lipid profile and POC urine test for microalbuminuria, retinal photography using non-mydriatic hand-held retinal camera, visual acuity assessment in both eyes and complete quality of life questionnaires. The primary aim of the study is to develop a model and assess its diagnostic performance to predict glycated haemoglobin (HbA1c) diagnosed diabetes from simple tests that can be applied in resource-limited settings; secondary outcomes include RBG cut-off for definition of pre-diabetes; diagnostic accuracy of cost-effective risk stratification models for diabetic retinopathy (DR); and models for identifying those at risk of complications of diabetes.

Diagnostic accuracy inter–tests agreement, statistical and economic modelling will be performed, accounting for clustering effects.

ETHICS AND DISSEMINATION:

The Indian Council of Medical Research (ICMR)/Health Ministry Screening Committee (HMSC) and Institutional Ethical Committees of all the participating Institutions approved the study (Ref: 2018-0494). Results will be published in peer-reviewed journals and will be presented at national and international conferences.

TRIAL REGISTRATION NUMBER: ISRCTN57962668

For peer review only

Article Summary

Strengths and Limitations of the study

1. This is the first national prospective study that will assess the prevalence of sight threatening diabetic retinopathy in various regions in India.
2. The study will provide evidence on the accuracy of point-of-care HbA1c as a screening tool for diabetes
3. The study will provide several diagnostic models on diabetes and its complications.
4. Validation of the models may not be possible in all cases.
5. The treatment pathway for patients identified with sight threatening diabetic retinopathy or other complications of diabetes is according to local protocols.

For peer review only

INTRODUCTION

Background

Diabetes and its complications are common causes of morbidity and mortality globally. Low and middle income countries (LMIC) are most affected by the diabetes epidemic, where significant number of people with undiagnosed diabetes present with complications of diabetes.[1] More than 30% of world population is estimated to have pre-diabetes.[2] The most common risk factors for diabetes and its complications are long term diabetes, uncontrolled hyperglycaemia, hypertension and dyslipidaemia. As high as 90% of people with type 2 diabetes are dyslipidaemic and 60-85% are hypertensive. In addition, 90% of people with type 2 diabetes are obese.[3] There is an unmet need to screen for pre-diabetes and diabetes in LMIC, where primary health care is under-developed and laboratory tests are costly.

Screening for people at risk of diabetes

According to the World Health Organisation (WHO), diabetes is confirmed by laboratory tests in a symptomatic individual if glycated haemoglobin (HbA1c) is ≥48mmol/L (≥6.5%) or fasting blood glucose is ≥7 mmol/L (≥126mg/dl), or a random blood glucose (RBG) is ≥11.1 mmol/L (≥200mg/dl) or after a 2-hour oral glucose tolerance test, blood glucose is ≥11.1 mmol/L (≥200mg/dl). In asymptomatic individuals, diabetes has to be confirmed by two of these laboratory tests.[4] Standard laboratory based HbA1c test have the added advantage of providing an average estimation of the glycaemic status of an individual over the previous 3 months and is helpful in categorising people into normal (HbA1c < 42mmol/mol; < 6.0%), prediabetes (HbA1c 42 to 47mmol/mol; 6 to 6.4%) and diabetes (HbA1c is ≥48mmol/mol; ≥6.5%). The lower limit of HbA1c in pre-diabetes may be as low as 5.7%. [5]

However, none of these tests are practical for population level screening in LMIC where non-technical personnel often conduct screening for diabetes in non-clinical environments. HbA1c also cannot be measured in patients with haemoglobinopathies. A number of LMIC have high prevalence of malaria and various haemoglobinopathies including thalassemia and sickle cell anaemia. Therefore, there is an unmet need to use simple tests to identify people at risk for diabetes. Despite its variability, capillary RBG is the commonest blood test done in such situations.[6] Pre-diabetes is not clearly defined by RBG. More convenient point-of-care (POC) HbA1c kits are now available that show good correlation with laboratory-based HbA1c estimation.[7] It is therefore appropriate to validate POC HbA1c against RBG in community screening.

Due to the large numbers of undiagnosed diabetes, it is also useful to investigate whether it is more efficient to triage people at risk of diabetes in the population using non-invasive diabetes risk scores, such as Madras Diabetes Research Foundation- Indian Diabetes Risk Score (MDRF-IDRS) [8] to further reduce the cost of screening with POC HbA1c or RBG.

Screening for complications of diabetes mellitus

For peer review only - http://bmjopen.bmj.com/site/about/guidelines.xhtml

Approximately 30% of people with diabetes present with macrovascular complications such as cardiovascular, cerebrovascular and peripheral vascular disease.[3] In addition, this population may also have microvascular complications including diabetic kidney disease (DKD) in 30-50%, diabetic retinopathy (DR) in 30% and diabetic neuropathy in 30-50%. [3] Despite this public health burden, people with diabetes are not systematically screened for these complications of diabetes in LMIC due to economic constraints, paucity of public health programmes, inadequately trained manpower and under-resourced infrastructure. Recently, several cardiovascular risk scores such as the non-laboratory INTERHEART risk score (NL-IHRS) have been successfully used in community screening programmes.[9] It may be possible to develop similar models to identify people at risk of sight-threatening diabetic retinopathy (STDR) and blindness. Although systematic annual photographic retinal screening after pupil dilatation using standard costly retinal cameras and prompt treatment of STDR have reduced the rate of blindness in the UK, [10] these complex and costly screening protocols are not translatable to LMIC and hence alternative screening methods must be considered to ensure population coverage. There are recent reports of accuracy of identifying STDR from the retinal images obtained by affordable and portable non-mydriatic cameras and graded either manually or by artificial intelligence. [11, 12] Therefore, adding retinopathy screening, using these hand-held retinal cameras, to minimally invasive tests, such as blood pressure (BP) and urine dip test for microalbuminuria and other non-laboratory (NL) risk scores may be an efficient and cost-effective screening option to identify people at risk of diabetes complications.

Objectives

Our study has three important objectives. The first objective is to determine the ideal tests that could identify people at risk of diabetes and pre-diabetes in community screening that can be applied to LMIC. In order to accomplish this, we would evaluate the correlation of RBG levels with POC HbA1c levels and decide on a cut-off value for RBG from HbA1c to diagnose pre-diabetes. Secondly, we will evaluate whether initial triaging with NL diabetes risk score followed by either RBG or POC HbA1c only to the identified risk-group is more effective than screening everyone for diabetes using either RBG or POC HbA1c. Thirdly, we will develop affordable, easily deliverable, and clinically effective model to accurately identify people at risk of complications of diabetes in community screening, especially DR. Secondary objectives are aimed at guiding future policies on screening of diabetes and its complications. As the study involves a large sample and the setting up of a teleophthalmology model to screen for DR across 20 regions in India, we will be able to report the regional prevalence of DR and the associated risk factors, the inter-grader reliability, and the accuracy of using artificial intelligence to grade DR. We will also conduct economic modelling and process evaluation of a holistic model for screening of all complications of diabetes. If sample size permits, we will be able to report on region-specific and diverse population specific rates of diabetes and complications, visual impairment, quality of life and risk models specific to regions to inform local health authorities.

METHODS AND ANALYSIS

For peer review only

Study design

This is a statistical and economic modelling study on a cross-sectional, prospective, cohort of participants recruited from community-based screening in order to accurately identify people at risk of diabetes, pre-diabetes and complications of diabetes.

Study setting

This community screening will be conducted across 20 regions in India, each led by a local clinical centre with a trained ophthalmologist responsible for the study at that site (table 1). Each region will have 3 clusters stratified into urban, rural and a pre-defined special category of population such as people with poor access to healthcare, or cohorts that are presumed high-risk or low-risk of developing diabetes. The study will involve a door-to-door survey, with questionnaires and POC tests performed by field workers. Each cluster will screen at least 800 consenting individuals aged 40 years or older for a cumulative sample size of a minimum of 48,000 participants. If any cluster or centre does not reach their target recruitment, it will be made up by another cluster or centre with the same stratified population.

Stratified sampling

In each region, we pre-defined a geographic area as urban or rural based on a multistage sampling technique using data from the 2011 census of India. A census enumeration block that usually consists of 125-150 households with a population of 650-700 is the primary sampling unit for urban areas while villages are defined in the rural areas. Bigger villages are further divided to ensure that approximately 300 households can be covered. The house-to-house survey will be conducted by approaching each household in consecutive streets in each area. If the household members are not available, a further 2 visits by the fieldworkers are permitted. In each household, all available members aged 40 years or above, who meet the inclusion criteria, will be invited to participate in the study.

The special category groups include two groups: (A) people working under high stress leading to poor and untimely eating habits, (such as policemen, truck and taxi drivers, manual labourers, fishermen, factory staff, professionals in stressful jobs) and those presumed to be of low risks such as certain religious groups and (B) people with poor health seeking behaviour and/or under social stigma (such as tribal, slum population, people with infection like human immunodeficiency virus or leprosy). All survey clusters and special groups are independent samples. The total population for the study is the total recruited participants in all the 20 regions including the special population (*Figure 1*).

Selection of participants

The inclusion criteria are adults who are ≥ 40 years of age (special groups may contain adult population of any age) who are local residents of Indian origin and are willing to give informed consent. Exclusion criteria include vulnerable adults in whom it may not be possible to carry out all the tests; pregnant and breast feeding women; anyone in the opinion of the fields worker deemed too ill to be screened; and those who are currently participating in intervention trials with investigational medicinal products.

For peer review only

Study procedures

The fieldworkers will be responsible for providing adequate information about the study and obtaining consent from willing participants. A unique patient identification number will be allocated for each participant to ensure anonymity. A detailed case report form containing a structured questionnaire will be answered by all participants in the study. The data collected will include age, gender, marital status, socioeconomic status (education, occupation, average monthly income), MDRF-IDRS and INTERHEART risk score that contain questions on lifestyle (smoking and alcohol habits, diet and physical activity and stress),[6, 7] brief medical and ocular history with any relevant medications and/or surgery, family history of diabetes and cardiovascular disease. The structured questionnaire will be translated into local languages and administered by trained field workers. Questionnaires will be validated in 200 subjects in 2 study sites at the start of the study and the case report forms and the study database will be refined to ensure generalisability and reproducibility.

Anthropometric measurements will be performed using the same kits supplied to all sites, and local field workers will be trained on regular calibration of the kits. Height (in centimetres) will be measured using a stadiometer (SECA Model 214, Seca Gmbh Co, Hamburg, Germany). Weight (in kilograms) will be measured with an electronic weighing scale (SECA Model 807, Seca Gmbh Co, Hamburg, Germany) kept on a firm horizontal flat surface. Body mass index will be auto-calculated. Waist circumference will be measured at the smallest horizontal girth between the costal margins and the iliac crest at the end of expiration using a non-stretchable measuring tape. Hip measurement will be done with the arms relaxed at the sides, at the maximum circumference over the buttocks.

Blood pressure (BP) will be recorded in sitting position in the right arm to the nearest 1 mm Hg using the electronic OMRON machine (Omron Corporation, Kyoto, Japan). Participants with BP≥ 140/90 mm Hg and not on antihypertensive drugs will be advised to contact a physician for further evaluation. A simple finger-prick test will be used to assess capillary RBG using a standard POC testing device (OneTouch Verio Glucometer, LifeScan Inc, United States). All participants with known diabetes or those with capillary RBG≥ 160mg/dl and 50 participants with RBG 110 to 159mg/dl in each cluster will receive further tests. These include HbA1c estimation using a POC kit (A1c Now Plus, PTS Diagnostics, United States) and POC lipid estimation (Cardiochek PA analyser, PTS Diagnostics, United States). A POC urine sample (Chemstrip Micral dipstick, Roche Diagnostics, Mannheim) will be tested for presence or absence of microalbuminuria.

Visual acuity in both eyes will be recorded using a tablet/smartphone-based vision check web-based application (Peek Vision). Non-mydriatic fundus photography of both eyes will be done using a handheld retinal camera (Visuscout 100, Zeiss, Germany). This portable and battery-operated camera with inbuilt wi-fi facilities will allow capture of colour and red free retinal images covering 40º field of view through pupils as small as 3.5mm. Two fundus images (one macula- and one disc- centred) of each eye will be captured. In case of any media opacities making fundus imaging difficult, the anterior segment image of each eye would be taken. A teleophthalmology system will be set up whereby the images captured by each field worker will be uploaded to a cloud-based study specific database and graded at the local clinical centre by an ophthalmologist / optometrist (primary grader), as well as

For peer review only

transferred to 4 central reading centres, where grading will be done by a second ophthalmologist (secondary grader). Discrepancies between primary and secondary grading will result in arbitration by a senior retinal consultant. Any participants with STDR, ungradable images and other incidental findings requiring further evaluation will be informed by the fieldworkers and counselled to attend hospital eyecare service. DR will be classified as per the International Clinical Disease Severity Scale for DR as no DR, mild / moderate / severe non–proliferative DR (NPDR) and proliferative DR (PDR). [13] Diabetic macular oedema [DMO] will be determined as present or absent. STDR would be defined as presence of severe NPDR, PDR and/or DMO. Artificial intelligence may be applied to grade these images and if found to be as accurate as human graders, it will be incorporated to the screening model.
The well-established and widely used quality of life questionnaire EQ-5D (Euro Quality of life) will also be administered with additional vision 'bolt-on' questions and vision related quality of life (VisQoL). [14-16] The study flow is shown in *Figure 2*. In addition, centre administrators at each clinical site will be responsible for contacting, by letter or phone, and tracking follow-up of those participants who need further referral to an eye hospital for treatment for STDR or due to ungradable retinal images.

Quality Assurance

Training of research personnel on study assessments will be done at study initiation meetings where the core study team, laboratory staff and camera manufacturers will certify individual field workers. In addition, the data manager in the UK will provide on-site training at each centre, as well as continuous remote training throughout the study. The ophthalmologists or their representatives at each clinical centre will be responsible for training their team who may not meet the pre-set criteria or any new member joining the team. A monitoring plan will be in place to ensure that regular remote monitoring is done throughout the study period.

Quality control

Calibration procedure and frequency for the weighing machine, BP apparatus, POC kits for capillary RBG and HbA1c and urine will be followed at all centres to avoid any bias or errors. All personnel involved in the grading of retinal images must have completed a study-specific training course.

Data management

The data will be entered directly by the field workers into a tablet that is linked to a cloud-based electronic database hosted in India. In situations where internet access is not available, paper case report forms will be used at the site and later transcribed into the database. The data in the database will be monitored by the study monitoring team. The retinal photographs will also be uploaded to the platform. The WHO STEPS (STEP wise approach to surveillance) approach will be used to develop the cloud-based electronic database.[17]

Database functionality and quality assurance

The study electronic database (Playon Ltd, Bangalore, India) will be hosted on a dedicated secure server in India. All data will be managed through this system. The database will be programmed to perform validation checks, such as range checks to prevent data entry errors,

For peer review only

missing data to be flagged up to ensure completion of the data entry. The system will provide for data security and also have formal database lock functionality and it will support real time data cleaning and reporting.

Statistical considerations

The statistical methods will be developed fully within a Statistical Analysis Plan, to be finalised before database lock. Diagnostic accuracy publications will follow recognised STARD (Standards for Reporting Diagnostic accuracy studies) guidelines and the observational component will follow the STROBE (Strengthening the Reporting of Observational studies in Epidemiology) guidelines. *Table 2* shows the reference and index tests for diagnostic accuracy aspect of the study.

Table 2: Reference and Index tests

**Community screening for diabetes**	
**Reference Standard**	**Index test**
1. RBG	1. POC HbA1c<br>2. Non-invasive diabetes risk scores
**Community screening for pre-diabetes**	
1. POC HbA1c	1. RBG<br>2. Non-invasive diabetes risk scores
**Community screening for complications of diabetes**	
1. Serum lipid profile<br>• Total Cholesterol (TC)<br>• Non HDL* Cholesterol<br>• LDL** Cholesterol<br>• HDL Cholesterol<br>• TC:HDL ratio<br>• Triglyceride	Risk-based screening tool for complications of diabetes utilising minimally or non-invasive tests.
2. HbA1c or RBG	
3. Microalbuminuria	
4. Retinal photography for retinopathy for all people with diabetes	

*HDL: High Density Lipoprotein **LDL: Low Density Lipoprotein

Accuracy will be measured by sensitivity and specificity of tests to detect diabetes, pre-diabetes and people at risk of complications of diabetes. Clustering will be used to accommodate any over dispersion. Consistency of these statistics will be explored across centres and clusters (urban, rural and special population). Area under ROC (Receiver

For peer review only

Operating Characteristic) curve will be used to compare models representing the overall performance of tests under comparison. Refinement of test components (e.g. combinations of tests, or questionnaire items) will be developed, and internally validated where sufficient data is available. The number of false positives will be identified directly from the data. From the estimates of sensitivity and the specificity of diabetes risk score to detect pre-diabetic (or diabetic) and its estimated prevalence, it will be possible to estimate the false positive rate and the complement of the positive predictive value. All estimates will be accompanied by estimated 95% confidence intervals, which account for both clustering and stratification.

For the modelling framework, a marginal model with a logit link will be used, with retinal photograph determination of the reference outcome. Model-predicted probabilities will enable the area under the ROC curve to be estimated with 95% confidence interval allowing for clustering, and accompanied by estimates of sensitivity, specificity, predictive values and likelihood ratios. Diabetes alone, and diabetes or pre-diabetes will be explored, as will already- and newly- identified diabetes. For research questions on the diabetes diagnostic model, the denominator will principally be all those diagnosed with diabetes, whether already- diagnosed or newly- diagnosed. Interaction with this term (known versus newly-diagnosed) will contribute to the analysis involving costs. Further modelling will explore use of the data from those that were found not to have diabetes or pre-diabetes.

Marginal logistic modelling will be used to identify the tests and questionnaire items which are most predictive, following a recommended approach.[18] Continuous predictors will be handled using the fractional polynomial approach.[19] In the sample size section it can be seen that the dataset is large enough to allow models to assess up to ten (reliably) and twenty (less reliably) dependent on intra-cluster correlation. Differences in area under ROC curve and differences in specificity for given sensitivity will be estimated. The sample size is large enough to assess existing tests and to develop models. There may be limited scope to validate models. However, interim analysis will allow assumed rates and numbers to be assessed; the number of cases with STDR will be estimated more accurately, and this may enable more sophisticated forms of internal validation. Model validation would include calibration after model discrimination.[20] Clustering within estimates of sensitivity, specificity, and areas under ROC curves will account for clustering, considering use of the nonparametric stratified bootstrap. A similar approach will be undertaken for the model to identify people at risk of complications of diabetes. Models for diabetic retinopathy will also test the accuracy of artificial intelligence graded images compared to human graders.

Sample size calculation

The sample size is determined by considering the numbers of expected STDR, as this analysis will have the smallest number of cases with the outcome. With 20 regions, we expect 216 cases of STDR. From 48,000 people (2,400 per centre) screened, of whom about 4,800 are expected to be known diabetes and, we suspect, another 4,800 will be newly detected diabetes. As 30% of the former group, and 15% of the latter group, are expected to have DR, we anticipated 2,160 people to have DR, of whom 216 to have STDR.

Considering that some patients would come from the same family, and some from the same area, we assumed that outcomes at the area level would have an allowed intra-centre

For peer review only

correlation (ICC) coefficients of approximately up to 0.05 and to 0.10 for new- and known-diabetes respectively. At the area level, with approximately 100 cases per region, and a working ICC of 0.075, we expect a design effect of 8.5. This calculation has been based on conservative allowances and approximations, which allow for deviations in the actual intra-cluster correlation coefficients from those anticipated, or for variation in the actual number of cases across centres. This means that the effective sample size (were the sample to be free from clustering) is 25 STDR cases for covariates, which are constant at the region level, or highly correlated amongst families within the same area. Using the rule of 10 people per covariate in order to plan the number of possible covariates, this implies that it will be possible to include 10 to 20 covariates (216/10) at the participant level dependent on whether there is no, modest, or moderately high ICC in the covariates, and 1 to 2 covariates (25/2) either at the area/family level for a stable diagnostic STDR model. All models will include observations at the participant level in order to accommodate participant-level covariates and will accommodate clustering further by including two area contrast terms; these reflect whether a participant lives in the strata of regions that are urban, rural or a special population. Models will be from the 'marginal' class so that correlation can be accommodated while importantly retaining a participant-specific interpretation of resulting estimates. The study will continue to recruit to enable process evaluation and other sub-studies to be incorporated.

Health economics analysis plan

The health economics modelling will address the following three questions; (1) What is the cost-effectiveness of a new screening pathway for diabetes and pre-diabetes? The screening approaches will comprise: diabetes risk score followed by definitive laboratory tests; diagnostic model which the statistical modelling finds to be more accurate than diabetes risk score followed by definitive laboratory tests; RBG for all without diabetes risk score based pre-screen; HbA1c test with no pre-screen; no screening; (2) What is the cost-effectiveness of a new screening pathway for DR among people with diabetes? The screening approaches will comprise a new method which the statistical modelling finds to be accurate; retinal photographs only; no screening. (3) What is the cost-effectiveness of a new screening pathway for a range of other complications of diabetes among people with diabetes? The screening approaches will comprise a new method, which the statistical modelling finds to be accurate; a combination of HbA1c, lipids and urine tests and colour retinal images; no screening. In each case therefore one comparator will be a 'gold standard' (HbA1c test, retinal photographs, combination of tests as above) and another will be no screening and no treatment until symptoms of DR, DKD or other complications of diabetes are experienced.

The modelling will draw on the following data sources: (1) The data collected through the house-to-house screening and associated retinal images, blood and urine tests on the rates of true and false positives and negatives, the characteristics of people with diabetes and its complications, and their quality of life. (2) Data collected through the study on the cost per person of this screening and its cost per person with diabetes, and the costs of clinic visits and treatments for DR. (3) Data and information from past studies on the incidence rates by age and gender of diabetes, DR and other complications of diabetes, transition rates between different stages of the disease, and disease-specific mortality rates. (4) Data from past studies on the costs of care for people with varying severities of DR and other complications of

For peer review only - http://bmjopen.bmj.com/site/about/guidelines.xhtml

diabetes and on their quality of life. For those variables on which data cannot be collected in this study or obtained from past studies, expert views will be sought, and sensitivity analyses conducted.

The modelling will comprise development of Markov models to track people from age 40 onward (a) through incidence of diabetes, any DR, STDR, severe visual impairment/blindness and (b) through incidence of diabetes, mild complications other than DR, and severe complications other than DR. For each disease state the models will contain estimates of average annual costs of care and average EQ5D quality of life. The design of the models will be developed in the light of data availability.

The models will be used to estimate lifetime costs and quality of life (monetised quality adjusted life years, QALYs) from age 40 and older (a) where the planned screening approach (or approaches) is conducted and necessary treatment given shortly after screening; (b) where the 'gold standard' screening approach is conducted and necessary treatment given shortly after screening; and (c) where no screening is conducted and no treatment given until symptoms develop. The incremental cost-effectiveness of the screening in comparison with 'gold standard' screening will be estimated by comparing (a) and (b); and its incremental cost-effectiveness in comparison with no screening will be estimated by comparing (a) and (c). A wide range of sensitivity analysis will be conducted, and a variety of discount rates may be applied.

We will also evaluate and compare the cost-effectiveness of retinal photography for everyone with diabetes versus retinal photography only for people with diabetes with suspected high risk of DR, to be developed through the statistical modelling. We will develop a health economics plan after reviewing available data. As an example, Rachapelle et al used a WHO recommended approach for a cost-effectiveness threshold in their study of the cost-utility of telemedicine to screen for DR in India.[21] Under that approach, the interventions costing less than per capita Gross Domestic Product (GDP) per QALY were considered very cost-effective, interventions between 1 and 3 times GDP were considered cost-effective and interventions more than 3 times GDP were considered not cost-effective.

Process Evaluation

A detailed process evaluation plan will be developed to evaluate the holistic screening for all complications of diabetes including the teleophthalmology. For each quantitative outcome measure, we will systematically embed qualitative measures in each RE-AIM dimension (reach, efficacy, adoption, implementation, maintenance) to evaluate the implementation strategy of community screening with minimally invasive tests. [22,23]

Outcomes:

The primary outcome is the correlation of RBG levels and POC HbA1c levels. Secondary outcomes include the cut-off value of RBG to define pre-diabetes; diagnostic accuracy of risk stratification models for diabetes; prevalence and risk stratification for screening for diabetic retinopathy; risk model for those at risk of complications of diabetes; identification of cost-effective diagnostic model for diabetes, pre-diabetes and complications of diabetes and

For peer review only

process evaluation of minimally invasive community screening for diabetes and its complications.

Ethics and dissemination

The Indian Council of Medical Research (ICMR) Health Ministry Screening Committee (HMSC) and the Institutional Ethical Committees of all the participating Institutions have approved the study. The main ethical issues in relation to this study are the identifications of people with risk factors for pre-diabetes, diabetes and its complications. However, the benefits of early diagnosis outweigh these risks. Participants who screen positive for any risk factors will be advised about referral to the local hospitals for treatment. Any breach of confidentiality will be minimised by anonymising participant identifiable information. The results will be published in Open Access peer reviewed journals, presented at scientific meetings and shared with the funder, and specific communication will be organised to target health professionals, policy decision-makers, regulatory bodies and commercial bodies for development of better predictive devices. The anonymised study data will be analysed by the statistical team in the UK.

References

1. IDF Diabetes Atlas, 9th edition 2019 https://www.diabetesatlas.org/en/
2. Hostalek U. Global epidemiology of prediabetes - present and future perspectives. *Clin Diabetes Endocrinol*2019; 5:5.
3. Bar-Tana J. Type 2 diabetes –unmet needs, unresolved pathogenesis, m-TORC1 centric paradigm *Rev Endocr Metab Disord*2020;1-17.
4. Classification of diabetes. World Health Organisation. 2019 www.who.int/publications-detail/classification-of-diabetes-mellitus.
5. Standards of Medical Care in Diabetes. *Diabetes Care*2019; 42(Suppl.1): S1-204.
6. Somannavar S, Ganesan A, Deepa M,et al. Random capillary blood glucose cut points for diabetes and pre-diabetes derived from community-based opportunistic screening in India. *Diabetes Care*2009;32(4):641–643.
7. Sicard DA1, Taylor JR Comparison of POC HbA1c test versus standardized laboratory testing. *Ann Pharmacother*2005;39(6):1024-8.
8. Mohan V, Anbalagan VP. Expanding role of the Madras Diabetes Research Foundation - Indian Diabetes Risk Score in clinical practice. *Indian J Endocrinol Metab*2013;17(1):31-6.
9. Joseph P, Yusuf S, Lee SF, et al. PURE INVESTIGATORS. Prognostic validation of a non-laboratory and a laboratory based cardiovascular disease risk score in multiple regions of the world. *Heart*2018:104(7):581-87.
10. Scanlon PH, Aldington SJ, Leal J, et al. Development of a cost-effectiveness model for optimisation of the screening interval in diabetic retinopathy screening. *Health Technol Assess*2015;19(74):1-116.
11. Natarajan S, Jain A, Krishnan R, et al. Diagnostic Accuracy of Community-Based Diabetic Retinopathy Screening With an Offline Artificial Intelligence System on a Smartphone. *JAMA Ophthalmol*2019:137(10);1182-88.
12. Rajalakshmi R, Arulmalar S, Usha M et al., Validation of Smartphone Based Retinal Photography for Diabetic Retinopathy Screening. *PLoS One*2015:10(9):e0138285.

For peer review only

13. Wilkinson CP, Ferris FL 3rd, Klein RE, et al. Global Diabetic Retinopathy Project Group. Proposed international clinical diabetic retinopathy and diabetic macular edema disease severity scales. *Ophthalmology*2003;110(9):1677-82.
14. Misajon R, Hawthorne G, Richardson J, et al. Vision and quality of life: the development of a utility measure. *Invest Ophthalmol Vis Sci*2005;46(11):4007-15.
15. Peacock S, Misajon R, Iezzi A, et al. Vision and quality of life:development of methods for the VisQoL vision-related utility instrument. *Ophthalmic Epidemiol*2008;15(4):218-23.
16. Janssen MF, Birnie E, Bonsel GJ. Quantification of the level descriptors for the standard EQ-5D three-level system and a five-level version according to two methods. *Qual Life Res*2008;17(3):463-73.
17. The WHO STEPwise approach to surveillance of noncommunicable diseases (STEPS). Noncommunicable Diseases and Mental Health. World Health Organization. 20 Avenue Appia, 1211 Geneva 27, Switzerland. http://www.who.int/ncd_surveillance
18. Royston P, Moons KGM, Altman DG, et al. Prognosis and prognostic research: Developing a prognostic model. *BMJ*2009;338:b604.
19. Sauerbrei W, Royston P. Building multivariable prognostic and diagnostic models: transformation of the predictors by using fractional polynomials. *J R Stat Soc Series A*1999;162:71-94
20. Altman DG, Vergouwe Y, Royston P, et al. Prognosis and prognostic research:validating a prognostic model. *BMJ*2009;338:b605
21. Rachapelle S, Legood R, Alavi Y, et al. The cost-utility of telemedicine to screen for diabetic retinopathy in India. *Ophthalmology*2013;120(3):566-73.
22. Moore GF, Audrey S, Barker M, et al., Process evaluation of complex interventions: Medical Research Council guidance. *BMJ*2015;350:h1258.
23. Forman J, Heisler M, Damschroder LJ, et al. Development and application of the RE-AIM QuEST mixed methods framework for program evaluation. *Prev Med Rep*2017; 6:322-28.

Legends for figures:

Figure 1: Map of India with 20 centres marked.

Figure 2: Study flow diagram

For peer review only

Author Statement

SMART India writing group: Sobha Sivaprasad (S.S), Rajiv Raman (R.R SN), Ramachandran Rajalakshmi (R.R MDRF), Viswanathan Mohan (V.M), Deepa Mohan (D.M), Taraprasad Das (T.D), Kim Ramasamy (K.R), Toby Prevost (T.P), Raphael Wittenberg (R.W), Gopal Netuveli (G.N), Gopal Lingam (G.L), Wasim Hanif (W.H), Dolores Conroy (D.C), Jayashree Ramu (J.R), Janani Surya (J.S) and Radha Ramakrishnan (R.R UCL).

Conceptualisation, S.S, R.R (SN), R.R (MDRF), V.M, K.R, D.M, J.R, T.P, G.L, T.D, G.N, R.W; methodology, S.S, T.D, G.N, R.W, T.P, J.R, D.M, R.R (UCL); formal analysis, S.S, G.N, R.W, T.P, R.R (UCL); writing—original draft preparation, S.S, D.C, R.R (SN), R.R (MDRF), T.D, G.N, R.W, J.S, W.H, J.R, T.P; writing—review and editing, S.S, R.R (SN), R.R (MDRF),

V.M, K.R, G.L, T.D, G.N, R.W, J.R, T.P, W.H, D.M, D.C, R.R (UCL); funding acquisition, S.S, R.R (SN), R.R (MDRF), V.M, K.R, T.D, G.N, R.W. on behalf of the SMART India Collaborators in Table 1.

Table 1: SMART-India Collaborators

Site No.	Name of Principal Investigator	Hospital Name
1	Dr Pramod Bhende<br>Dr Rajiv Raman	Sankara Nethralaya, Chennai, Tamil Nadu
2	Dr Ramachandran Rajalakshmi<br>Dr Viswanathan Mohan	Dr Mohan's Diabetes Specialities Centre, Chennai, Tamil Nadu
3	Dr Kim Ramasamy	Aravind Eye Hospital, Madurai, Tamil Nadu
4	Dr Taraprasad Das<br>Dr Padmaja K Rani	LV Prasad Eye Institute, Hyderabad, Telangana
5	Dr Rupak Roy<br>Dr Supita Das	Sankara Nethralaya, Kolkata
6	Dr Deepa Mohan	Dr Mohan's Diabetes Specialities Centre, Mysuru, Karnataka
7	Dr V Narendran<br>Dr George Manayath	Aravind Eye Hospital, Coimbatore, Tamil Nadu
8	Dr Giridhar Anantharaman<br>Dr Mahesh Gopalakrishnan	Giridhar Eye Institute, Cochin, Kerala
9	Dr Sundaram Natarajan<br>Dr Radhika Krishnan	Aditya Jyot Hospital, Mumbai, Maharashtra
10	Dr Sheena Liz Mani	Dr Tony Fernandez Eye Hospital, Aluva, Kerala
11	Dr Manisha Agarwal	Dr Shroff's Charity Eye Hospital, New Delhi
12	Dr Tapas Padhi<br>Dr Umesh Behera	LV Prasad Eye Institute, Bhubaneshwar, Odisha
13	Dr Harsha Bhattacharjee<br>Dr Manabjyoti Barman	Sri Sankaradeva Nethralaya, Guwahati, Assam
14	Dr Gajendra Chawla	Vision Academy- The Socio Medical Society, Bhopal, Madhya Pradesh
15	Dr Alok Sen	Sadguru Netra Chikitsalaya, Chitrakoot, Madhya Pradesh
16	Dr Moneesh Saxena	Aurobindo Nethralaya, Raipur, Chhattisgarh
17	Dr Asim K Sil<br>Dr Subhratanu Chakabarty	Netra Niramay Niketan, Haldia, West Bengal
18	Dr Thomas Cherian<br>Dr Reesha KR	Little Flower Hospital & Research Center, Angamaly, Kerala
19	Dr Rushikesh Naigaonkar<br>Dr Abishek Desai	Ganapathy Nethralaya, Jalna, Maharashtra
20	Dr Col Madan Deshpande<br>Dr Sucheta Kulkarni	HV Desai Hospital, Pune, Maharashtra

For peer review only

Acknowledgements: The authors would like to thank all the Study collaborators including field workers, each centre staff, reading centre staff, Jitendra Pal Thethi for the study database; and the study participants.

Patient and Public Involvement: Patients or the public were not involved in the design, or conduct, or reporting, or dissemination plans of our research

Funding statement: This work was supported by Global Challenges Research Fund and UK Research and Innovation through the Medical Research Council grant number MR/P027881/1

Data Sharing Agreement: The technical appendix, statistical code and dataset will be made available on request after review by the Study Group.

Competing interests statement: None

For peer review only

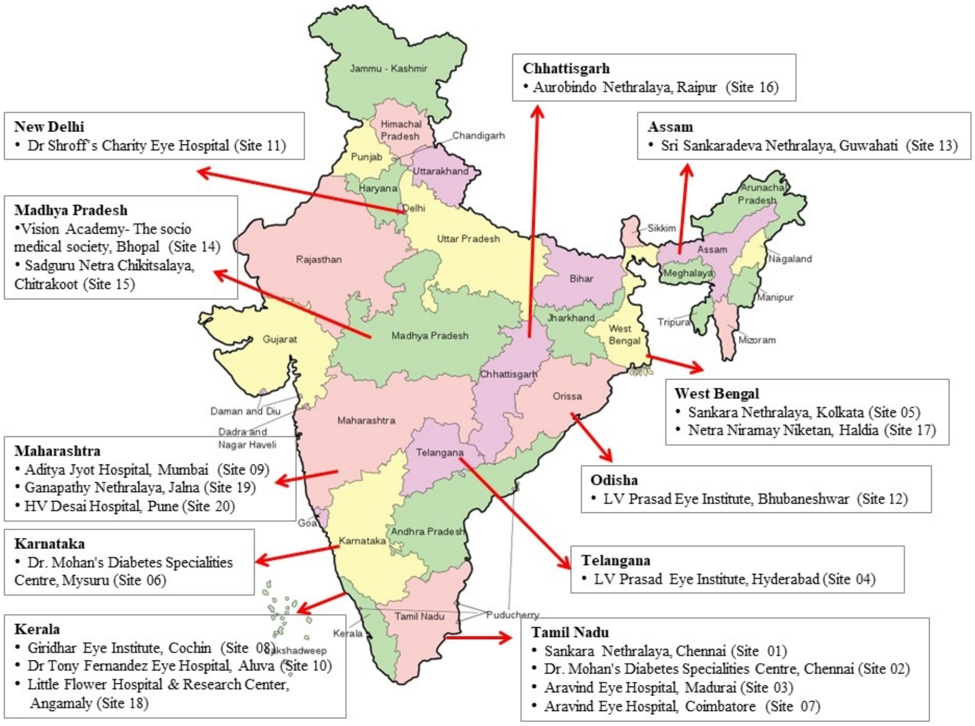

Figure 1: Map of India with 20 centres marked

81x60mm (300 x 300 DPI)

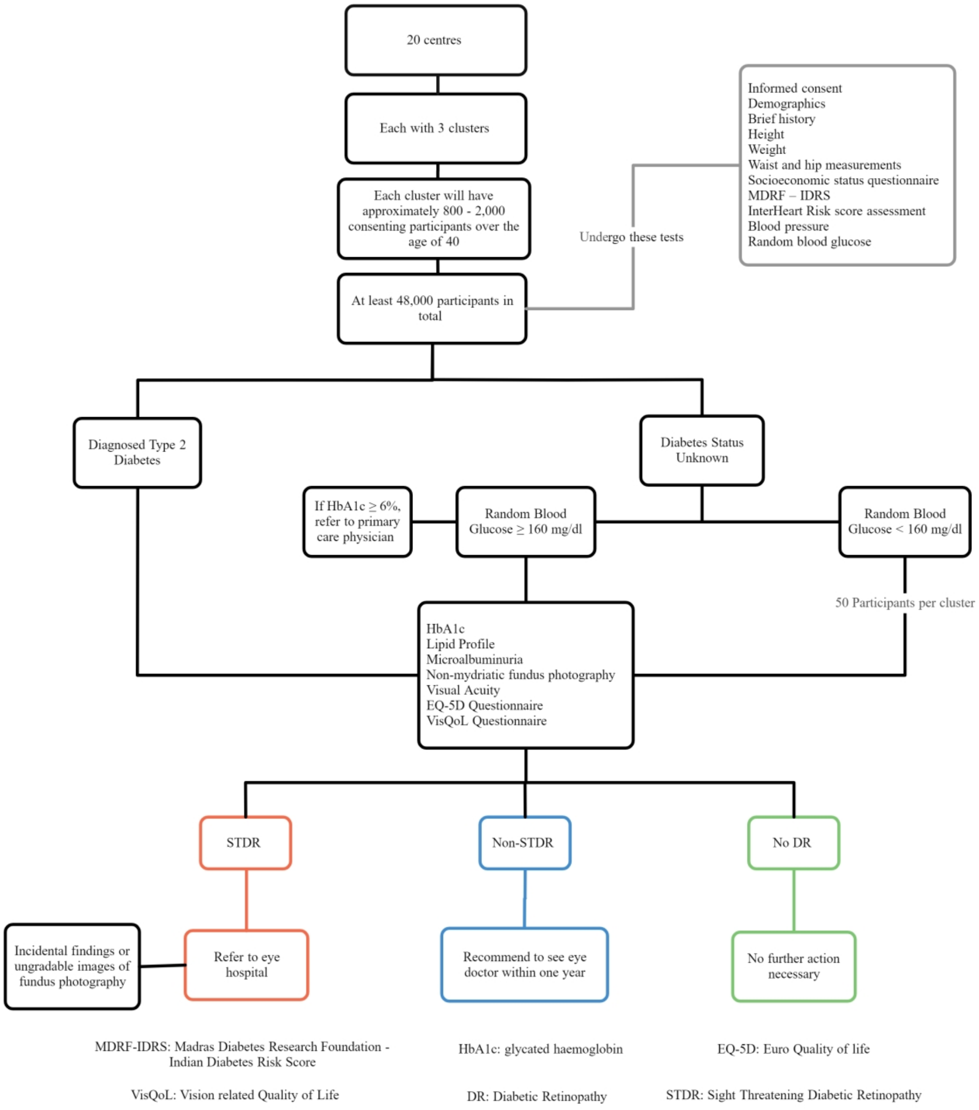

Figure 2: Study flow diagram

BMJ Open

Protocol on a multi-centre statistical and economic modelling study of risk-based stratified and personalised screening for diabetes and its complications in India (SMART India).

Journal:	*BMJ Open*
Manuscript ID	bmjopen-2020-039657.R1
Article Type:	Protocol
Date Submitted by the Author:	05-Aug-2020
Complete List of Authors:	Sivaprasad, Sobha; Moorfields Eye Hospital NHS Foundation Trust, Medical retina<br>Raman, Rajiv; Sankara Nethralaya, Shri Bhagwan Mahavir Vitreoretinal services<br>Rajalakshmi, Ramachandran; Dr Mohans Diabet Special Ctr, Ophthalmology; DR. MOHAN'S DIABETES SPECIALITIES CENTRE AND MADRAS DIABETES RESEARCH FOUNDATION<br>Mohan, V; Madras Diabetes Research Foundation<br>Deepa, Mohan; Madras Diabetes Research Foundation and Dr. Mohan's Diabetes Specialities Centre<br>Das, Taraprasad; 5. Hyderabad Eye Research Foundation, L V Prasad Eye Institute<br>Ramasamy, Kim; Aravind Medical Research Foundation<br>Prevost, A Toby; Imperial College London, Imperial Clinical Trials Unit<br>Wittenberg, Raphael; University of Oxford, Centre for Health Service Economics and Organisation<br>Netuveli, Gopalakrishnan; University of East London, School of Health, Sports and Bioscience<br>Lingam, Gopal; National University Hospital<br>Hanif, Wasim; University Hospital Birmingham<br>Ramakrishnan, Radha; UCL Institute of Ophthalmology<br>Ramu, Jayashree; NIHR Moorfields Biomedical Research Centre, Medical Retina<br>Surya, Janani; Vision Research Foundation, Sankara Nethralaya<br>Conroy, Dolores; UCL Institute of Ophthalmology
**Primary Subject Heading**:	Diabetes and endocrinology
Secondary Subject Heading:	Epidemiology
Keywords:	Diabetic retinopathy < DIABETES & ENDOCRINOLOGY, General diabetes < DIABETES & ENDOCRINOLOGY, HEALTH ECONOMICS, Medical retina < OPHTHALMOLOGY, Epidemiology < TROPICAL MEDICINE, PUBLIC HEALTH

I, the Submitting Author has the right to grant and does grant on behalf of all authors of the Work (as defined in the below author licence), an exclusive licence and/or a non-exclusive licence for contributions from authors who are: i) UK Crown employees; ii) where BMJ has agreed a CC-BY licence shall apply, and/or iii) in accordance with the terms applicable for US Federal Government officers or employees acting as part of their official duties; on a worldwide, perpetual, irrevocable, royalty-free basis to BMJ Publishing Group Ltd ("BMJ") its licensees and where the relevant Journal is co-owned by BMJ to the co-owners of the Journal, to publish the Work in this journal and any other BMJ products and to exploit all rights, as set out in our licence.

The Submitting Author accepts and understands that any supply made under these terms is made by BMJ to the Submitting Author unless you are acting as an employee on behalf of your employer or a postgraduate student of an affiliated institution which is paying any applicable article publishing charge ("APC") for Open Access articles. Where the Submitting Author wishes to make the Work available on an Open Access basis (and intends to pay the relevant APC), the terms of reuse of such Open Access shall be governed by a Creative Commons licence – details of these licences and which Creative Commons licence will apply to this Work are set out in our licence referred to above.

Other than as permitted in any relevant BMJ Author's Self Archiving Policies, I confirm this Work has not been accepted for publication elsewhere, is not being considered for publication elsewhere and does not duplicate material already published. I confirm all authors consent to publication of this Work and authorise the granting of this licence.

For peer review only

Protocol on a multi-centre statistical and economic modelling study of risk-based stratified and personalised screening for diabetes and its complications in India (SMART India).

Sobha Sivaprasad[1, 2], Rajiv Raman[3], Ramachandran Rajalakshmi[4], Viswanathan Mohan[4], Deepa Mohan[4], Taraprasad Das[5], Kim Ramasamy[6], Toby Prevost[7], Raphael Wittenberg[8], Gopal Netuveli,[9] Gopal Lingam[10], Wasim Hanif[11], Radha Ramakrishnan[2], Jayashree Ramu[1], Janani Surya[3] and Dolores Conroy[2] for the SMART India Study Collaborators

1. NIHR Biomedical Research Centre, Moorfields Eye Hospital NHS Foundation Trust, London, UK
2. UCL Institute of Ophthalmology, London, UK
3. Vision Research Foundation, Sankara Nethralaya, Chennai, India
4. Madras Diabetes Research Foundation and Dr Mohan's Diabetes Specialities Centre, Chennai, India
5. Hyderabad Eye Research Foundation, L V Prasad Eye Institute, Hyderabad, India
6. Aravind Medical Research Foundation, Madurai, India
7. Imperial College London, UK
8. University of Oxford, UK
9. University of East London, UK
10. National University Hospital, Singapore
11. University Hospital, Birmingham, UK

Word count: 4450

Tables: 2

Figures: 2

References: 33

Abstract: 276

Corresponding author

Professor Sobha Sivaprasad
NIHR Moorfields Biomedical Research Centre
Moorfields Eye Hospital NHS Foundation Trust,
162 City Road,
London EC1V 2PD, UK
Tel: 020 7253 3411
Email: senswathi@aol.com

Keywords: Diabetes, glycated haemoglobin, diabetic retinopathy, diabetes complications, India

Abstract

For peer review only

INTRODUCTION:
The aim of this study is to develop practical and affordable models to (a) diagnose people with diabetes and pre-diabetes and (b) identify those at risk of diabetes complications so that these models can be applied to the population in low and middle-income countries (LMIC) where laboratory tests are unaffordable.

METHODS AND ANALYSIS:
This statistical and economic modelling study will be done on at least 48,000 prospectively recruited participants aged 40 years or above through community screening across 20 pre-defined regions in India. Each participant will be tested for capillary random blood glucose (RBG) and complete a detailed health related questionnaire. People with known diabetes and all participants with pre-defined levels of RBG will undergo further tests including point-of-care (POC) glycated haemoglobin (HbA1c), POC lipid profile and POC urine test for microalbuminuria, retinal photography using non-mydriatic hand-held retinal camera, visual acuity assessment in both eyes and complete quality of life questionnaires. The primary aim of the study is to develop a model and assess its diagnostic performance to predict glycated haemoglobin (HbA1c) diagnosed diabetes from simple tests that can be applied in resource-limited settings; secondary outcomes include RBG cut-off for definition of pre-diabetes; diagnostic accuracy of cost-effective risk stratification models for diabetic retinopathy (DR); and models for identifying those at risk of complications of diabetes.

Diagnostic accuracy inter–tests agreement, statistical and economic modelling will be performed, accounting for clustering effects.

ETHICS AND DISSEMINATION:

The Indian Council of Medical Research (ICMR)/Health Ministry Screening Committee (HMSC/2018-0494 dated 17/12/2018 and Institutional Ethics Committees of all the participating Institutions approved the study. Results will be published in peer-reviewed journals and will be presented at national and international conferences.

TRIAL REGISTRATION NUMBER: ISRCTN57962668 V1.0 24/09/2018

TRIAL SPONSOR: Vision Research Foundation, Sankara Nethralaya, 41 College Road, Chennai, Tamil Nadu, India

For peer review only

Article Summary

Strengths and Limitations of the study

1. This is the first national prospective study that will assess the prevalence of sight threatening diabetic retinopathy in various regions in India.
2. The study will provide evidence on the accuracy of point-of-care HbA1c as a screening tool for diabetes
3. The study will provide several diagnostic models on diabetes and its complications.
4. Validation of the models may not be possible in all cases.
5. The treatment pathway for patients identified with sight threatening diabetic retinopathy or other complications of diabetes is according to local protocols.

For peer review only

INTRODUCTION

Background

Diabetes and its complications are common causes of morbidity and mortality globally. Low and middle income countries (LMIC) are most affected by the diabetes epidemic, where significant number of people with undiagnosed diabetes present with complications of diabetes.[1] More than 30% of world population is estimated to have pre-diabetes.[2] The most common risk factors for diabetes and its complications are long term diabetes, uncontrolled hyperglycaemia, hypertension and dyslipidaemia. As high as 90% of people with type 2 diabetes are dyslipidaemic and 60-85% are hypertensive. In addition, 90% of people with type 2 diabetes are obese.[3] There is an unmet need to screen for pre-diabetes and diabetes in LMIC, where primary health care is under-developed and laboratory tests are costly.

Screening for people at risk of diabetes

According to the World Health Organisation (WHO), diabetes is confirmed by laboratory tests in a symptomatic individual if glycated haemoglobin (HbA1c) is ≥48mmol/L (≥6.5%) or fasting blood glucose is ≥7 mmol/L (≥126mg/dl), or a random blood glucose (RBG) is ≥11.1 mmol/L (≥200mg/dl) or after a 2-hour oral glucose tolerance test, blood glucose is ≥11.1 mmol/L (≥200mg/dl). In asymptomatic individuals, diabetes has to be confirmed by two of these laboratory tests.[4] Standard laboratory based HbA1c test have the added advantage of providing an average estimation of the glycaemic status of an individual over the previous 3 months and is helpful in categorising people into normal (HbA1c < 42mmol/mol; < 6.0%), prediabetes (HbA1c 42 to 47mmol/mol; 6 to 6.4%) and diabetes (HbA1c is ≥48mmol/mol; ≥6.5%).[4] The lower limit of HbA1c in pre-diabetes may be as low as 5.7%. [5]

However, none of these tests are practical for population level screening in LMIC where non-technical personnel often conduct screening for diabetes in non-clinical environments. HbA1c also cannot be measured in patients with haemoglobinopathies. A number of LMIC have high prevalence of malaria and various haemoglobinopathies including thalassemia and sickle cell anaemia. Therefore, there is an unmet need to use simple tests to identify people at risk for diabetes. Despite its variability, capillary RBG is the commonest blood test done in such situations.[6] Pre-diabetes is not clearly defined by RBG despite several studies that have attempted to define cut-off values of RBG against HbA1c.[6-15] More convenient point-of-care (POC) HbA1c kits are now available that show good correlation with laboratory-based HbA1c estimation.[16] It is therefore appropriate to validate POC HbA1c against RBG in community screening. Although there are several studies that have evaluated various screening tests for pre-diabetes, these studies have used laboratory-based HbA1c measurements or fasting blood glucose as the index test.[17] In contrast this study will focus on POC HbA1c as the index test for pre-diabetes to inform community screening. Studies using POC HbA1c as a reference test have included specific disease cohorts only, or had a small sample size within hospital settings or conducted post-hoc analysis on previously recruited study cohorts and most importantly, did not compare the accuracy of these tests with known non-laboratory based diabetes risk scores.[6-15]

For peer review only - http://bmjopen.bmj.com/site/about/guidelines.xhtml

Due to the large numbers of undiagnosed diabetes, it is also useful to investigate whether it is more efficient to triage people at risk of diabetes in the population using non-invasive diabetes risk scores, such as Madras Diabetes Research Foundation- Indian Diabetes Risk Score (MDRF-IDRS) [18] to further reduce the cost of screening with POC HbA1c or RBG.

Screening for complications of diabetes mellitus

Approximately 30% of people with diabetes present with macrovascular complications such as cardiovascular, cerebrovascular and peripheral vascular disease.[3] In addition, this population may also have microvascular complications including diabetic kidney disease (DKD) in 30-50%, diabetic retinopathy (DR) in 30% and diabetic neuropathy in 30-50%. [3] Despite this public health burden, people with diabetes are not systematically screened for these complications of diabetes in LMIC due to economic constraints, paucity of public health programmes, inadequately trained manpower and under-resourced infrastructure. Recently, several cardiovascular risk scores such as the non-laboratory INTERHEART risk score (NL-IHRS) have been successfully used in community screening programmes.[19] It may be possible to develop similar models to identify people at risk of sight-threatening diabetic retinopathy (STDR) and blindness. Although systematic annual photographic retinal screening after pupil dilatation using standard costly retinal cameras and prompt treatment of STDR have reduced the rate of blindness in the UK, [20] these complex and costly screening protocols are not translatable to LMIC and hence alternative screening methods must be considered to ensure population coverage. There are recent reports of accuracy of identifying STDR from the retinal images obtained by affordable and portable non-mydriatic cameras and graded either manually or by artificial intelligence. [21, 22] Therefore, adding retinopathy screening, using these hand-held retinal cameras, to minimally invasive tests, such as blood pressure (BP) and urine dip test for microalbuminuria and other non-laboratory (NL) risk scores may be an efficient and cost-effective screening option to identify people at risk of diabetes complications.

Objectives

Our study has three important objectives. The first objective is to determine the ideal tests that could identify people at risk of diabetes and pre-diabetes in community screening that can be applied to LMIC. In order to accomplish this, we would evaluate the correlation of RBG levels with POC HbA1c levels and decide on a cut-off value for RBG from HbA1c to diagnose pre-diabetes. Secondly, we will evaluate whether initial triaging with NL diabetes risk score followed by either RBG or POC HbA1c only to the identified risk-group is more effective than screening everyone for diabetes using either RBG or POC HbA1c. Thirdly, we will develop affordable, easily deliverable, and clinically effective model to accurately identify people at risk of complications of diabetes in community screening, especially DR. Secondary objectives are aimed at guiding future policies on screening of diabetes and its complications. As the study involves a large sample and the setting up of a teleophthalmology model to screen for DR across 20 regions in India, we will be able to report the regional prevalence of DR and the associated risk factors, the inter-grader reliability, and the accuracy of using artificial intelligence to grade DR. We will also conduct economic modelling and process evaluation of a holistic model for screening of all

For peer review only

complications of diabetes. If sample size permits, we will be able to report on region-specific and diverse population specific rates of diabetes and complications, visual impairment, quality of life and risk models specific to regions to inform local health authorities.

METHODS AND ANALYSIS

Study design

This is a statistical and economic modelling study that will be done on a cross-sectional and prospectively recruited participants from community-based screening in order to accurately identify people at risk of diabetes, pre-diabetes and complications of diabetes.

Study setting

This community screening will be conducted across 20 regions in India, each led by a local clinical centre with a trained ophthalmologist responsible for the study at that site (table 1). Each region will have 3 clusters stratified into urban, rural and a pre-defined special category of population such as people with poor access to healthcare, or persons that are presumed high-risk or low-risk of developing diabetes. The study will involve a door-to-door survey, with questionnaires and POC tests performed by field workers. Each cluster will screen at least 800 consenting individuals aged 40 years or older for a cumulative sample size of a minimum of 48,000 participants. If any cluster or centre does not reach their target recruitment, it will be made up by another cluster or centre with the same stratified population.

Stratified sampling

In each region, we pre-defined a geographic area as urban or rural based on a multistage sampling technique using data from the 2011 census of India. A census enumeration block that usually consists of 125-150 households with a population of 650-700 is the primary sampling unit for urban areas while villages are defined in the rural areas. Bigger villages are further divided to ensure that approximately 300 households can be covered. The house-to-house survey will be conducted by approaching each household in consecutive streets in each area. If the household members are not available, a further 2 visits by the fieldworkers are permitted. In each household, all available members aged 40 years or above, who meet the inclusion criteria, will be invited to participate in the study.

The special category groups include two groups: (A) people working under high stress leading to poor and untimely eating habits, (such as policemen, truck and taxi drivers, manual labourers, fishermen, factory staff, professionals in stressful jobs) and those presumed to be of low risks such as certain religious groups and (B) people with poor health seeking behaviour and/or under social stigma (such as tribal, slum population, people with infection like human immunodeficiency virus or leprosy). All survey clusters and special groups are independent samples. The total population for the study is the total recruited participants in all the 20 regions including the special population (*Figure 1*).

Selection of participants

The inclusion criteria are adults who are ≥ 40 years of age (special groups may contain adult

For peer review only

population of any age) who are local residents of Indian origin and are willing to give informed consent (see Appendix 1 for sample Informed Consent Form).
Exclusion criteria include vulnerable adults in whom it may not be possible to carry out all the tests; pregnant and breast feeding women; anyone in the opinion of the fields worker deemed too ill to be screened; and those who are currently participating in intervention trials with investigational medicinal products.

Study procedures

The fieldworkers will be responsible for providing adequate information about the study and obtaining consent from willing participants. A unique patient identification number will be allocated for each participant to ensure anonymity. A detailed case report form containing a structured questionnaire will be answered by all participants in the study (see Appendix 2 for case report form). The data collected will include age, gender, marital status, socioeconomic status (education, occupation, average monthly income), MDRF-IDRS and INTERHEART risk score that contain questions on lifestyle (smoking and alcohol habits, diet and physical activity and stress),[6, 7] brief medical and ocular history with any relevant medications and/or surgery, family history of diabetes and cardiovascular disease. The structured questionnaire will be translated into local languages and administered by trained field workers. Questionnaires will be validated in 200 subjects in 2 study sites at the start of the study and the case report forms and the study database will be refined to ensure generalisability and reproducibility.
Anthropometric measurements will be performed using the same kits supplied to all sites, and local field workers will be trained on regular calibration of the kits. Height (in centimetres) will be measured using a stadiometer (SECA Model 214, Seca Gmbh Co, Hamburg, Germany). Weight (in kilograms) will be measured with an electronic weighing scale (SECA Model 807, Seca Gmbh Co, Hamburg, Germany) kept on a firm horizontal flat surface. Body mass index will be auto-calculated. Waist circumference will be measured at the smallest horizontal girth between the costal margins and the iliac crest at the end of expiration using a non-stretchable measuring tape. Hip measurement will be done with the arms relaxed at the sides, at the maximum circumference over the buttocks.

Blood pressure (BP) will be recorded in sitting position in the right arm to the nearest 1 mm Hg using the electronic OMRON machine (Omron Corporation, Kyoto, Japan). Participants with BP≥ 140/90 mm Hg and not on antihypertensive drugs will be advised to contact a physician for further evaluation. A simple finger-prick test will be used to assess capillary RBG using a standard POC testing device (OneTouch Verio Glucometer, LifeScan Inc, United States). All participants with known diabetes or those with capillary RBG≥ 160mg/dl and 50 participants with RBG 110 to 159mg/dl in each cluster will receive further tests. These include HbA1c estimation using a POC kit (A1c Now Plus, PTS Diagnostics, United States) and POC lipid estimation (Cardiochek PA analyser, PTS Diagnostics, United States). A POC urine sample (Chemstrip Micral dipstick, Roche Diagnostics, Mannheim) will be tested for presence or absence of microalbuminuria.

Visual acuity in both eyes will be recorded using a tablet/smartphone-based vision check web-based application (Peek Vision). Non-mydriatic fundus photography of both eyes will be

For peer review only

done using a handheld retinal camera (Visuscout 100, Zeiss, Germany). This portable and battery-operated camera with inbuilt wi-fi facilities will allow capture of colour and red free retinal images covering 40º field of view through pupils as small as 3.5mm. Two fundus images (one macula- and one disc- centred) of each eye will be captured. In case of any media opacities making fundus imaging difficult, the anterior segment image of each eye would be taken. A teleophthalmology system will be set up whereby the images captured by each field worker will be uploaded to a cloud-based study specific database and graded at the local clinical centre by an ophthalmologist / optometrist (primary grader), as well as transferred to 4 central reading centres, where grading will be done by a second ophthalmologist (secondary grader). Discrepancies between primary and secondary grading will result in arbitration by a senior retinal consultant. Any participants with STDR, ungradable images and other incidental findings requiring further evaluation will be informed by the fieldworkers and counselled to attend hospital eyecare service. DR will be classified as per the International Clinical Disease Severity Scale for DR as no DR, mild / moderate / severe non–proliferative DR (NPDR) and proliferative DR (PDR). [23] Diabetic macular oedema [DMO] will be determined as present or absent. STDR would be defined as presence of severe NPDR, PDR and/or DMO. Artificial intelligence may be applied to grade these images and if found to be as accurate as human graders, it will be incorporated to the screening model.

The well-established and widely used quality of life questionnaire EQ-5D (Euro Quality of life) will also be administered with additional vision 'bolt-on' questions and vision related quality of life (VisQoL). [24-26] The study flow is shown in *Figure 2*. In addition, centre administrators at each clinical site will be responsible for contacting, by letter or phone, and tracking follow-up of those participants who need further referral to an eye hospital for treatment for STDR or due to ungradable retinal images.

Quality Assurance

Training of research personnel on study assessments will be done at study initiation meetings where the core study team, laboratory staff and camera manufacturers will certify individual field workers. In addition, the data manager in the UK will provide on-site training at each centre, as well as continuous remote training throughout the study. The ophthalmologists or their representatives at each clinical centre will be responsible for training their team who may not meet the pre-set criteria or any new member joining the team. A monitoring plan will be in place to ensure that regular remote monitoring is done throughout the study period.

Quality control

Calibration procedure and frequency for the weighing machine, BP apparatus, POC kits for capillary RBG and HbA1c and urine will be followed at all centres to avoid any bias or errors. All personnel involved in the grading of retinal images must have completed a study-specific training course.

Data management

The data will be entered directly by the field workers into a tablet that is linked to a cloud-based electronic database hosted in India. In situations where internet access is not available, paper case report forms will be used at the site and later transcribed into the database. The

For peer review only

data in the database will be monitored by the study monitoring team. The retinal photographs will also be uploaded to the platform. The WHO STEPS (STEP wise approach to surveillance) approach will be used to develop the cloud-based electronic database.[27] The study is monitored by an independent committee and the progress of the study is reviewed by the Grant Executive Committee.

Database functionality and quality assurance

The study electronic database (Playon Ltd, Bangalore, India) will be hosted on a dedicated secure server in India. All data will be managed through this system. The database will be programmed to perform validation checks, such as range checks to prevent data entry errors, missing data to be flagged up to ensure completion of the data entry. The system will provide for data security and also have formal database lock functionality and it will support real time data cleaning and reporting.

Statistical considerations

The statistical methods will be developed fully within a Statistical Analysis Plan, to be finalised before database lock. Diagnostic accuracy publications will follow recognised STARD (Standards for Reporting Diagnostic accuracy studies) guidelines and the observational component will follow the STROBE (Strengthening the Reporting of Observational studies in Epidemiology) guidelines. *Table 2* shows the reference and index tests for diagnostic accuracy aspect of the study.

Table 1: Reference and Index tests

**Community screening for diabetes**	
**Reference Standard**	**Index test**
1. RBG	1. POC HbA1c<br>2. Non-invasive diabetes risk scores
**Community screening for pre-diabetes**	
1. POC HbA1c	1. RBG<br>2. Non-invasive diabetes risk scores
**Community screening for complications of diabetes**	
1. Serum lipid profile<br>• Total Cholesterol (TC)<br>• Non HDL* Cholesterol<br>• LDL** Cholesterol<br>• HDL Cholesterol<br>• TC:HDL ratio<br>• Triglyceride	Risk-based screening tool for complications of diabetes utilising minimally or non-invasive tests.
2. HbA1c or RBG	

3. Microalbuminuria	
4. Retinal photography for retinopathy for all people with diabetes	

*HDL: High Density Lipoprotein **LDL: Low Density Lipoprotein

Accuracy will be measured by sensitivity and specificity of tests to detect diabetes, pre-diabetes and people at risk of complications of diabetes. Clustering will be used to accommodate any over dispersion. Consistency of these statistics will be explored across centres and clusters (urban, rural and special population). Area under ROC (Receiver Operating Characteristic) curve will be used to compare models representing the overall performance of tests under comparison. Refinement of test components (e.g. combinations of tests, or questionnaire items) will be developed, and internally validated where sufficient data is available. The number of false positives will be identified directly from the data. From the estimates of sensitivity and the specificity of diabetes risk score to detect pre-diabetic (or diabetic) and its estimated prevalence, it will be possible to estimate the false positive rate and the complement of the positive predictive value. All estimates will be accompanied by estimated 95% confidence intervals, which account for both clustering and stratification.

For the modelling framework, a marginal model with a logit link will be used, with retinal photograph determination of the reference outcome. Model-predicted probabilities will enable the area under the ROC curve to be estimated with 95% confidence interval allowing for clustering, and accompanied by estimates of sensitivity, specificity, predictive values and likelihood ratios. Diabetes alone, and diabetes or pre-diabetes will be explored, as will already- and newly- identified diabetes. For research questions on the diabetes diagnostic model, the denominator will principally be all those diagnosed with diabetes, whether already- diagnosed or newly- diagnosed. Interaction with this term (known versus newly-diagnosed) will contribute to the analysis involving costs. Further modelling will explore use of the data from those that were found not to have diabetes or pre-diabetes.

Marginal logistic modelling will be used to identify the tests and questionnaire items which are most predictive, following a recommended approach.[28] Continuous predictors will be handled using the fractional polynomial approach.[29] In the sample size section it can be seen that the dataset is large enough to allow models to assess up to ten (reliably) and twenty (less reliably) dependent on intra-cluster correlation. Differences in area under ROC curve and differences in specificity for given sensitivity will be estimated. The sample size is large enough to assess existing tests and to develop models. There may be limited scope to validate models. However, interim analysis will allow assumed rates and numbers to be assessed; the number of cases with STDR will be estimated more accurately, and this may enable more sophisticated forms of internal validation. Model validation would include calibration after model discrimination.[30] Clustering within estimates of sensitivity, specificity, and areas under ROC curves will account for clustering, considering use of the nonparametric stratified bootstrap. A similar approach will be undertaken for the model to identify people at risk of

For peer review only

complications of diabetes. Models for diabetic retinopathy will also test the accuracy of artificial intelligence graded images compared to human graders.

Sample size calculation

The sample size is determined by considering the numbers of expected STDR, as this analysis will have the smallest number of cases with the outcome. With 20 regions, we expect 216 cases of STDR. From 48,000 people (2,400 per centre) screened, of whom about 4,800 are expected to be known diabetes and, we suspect, another 4,800 will be newly detected diabetes. As 30% of the former group, and 15% of the latter group, are expected to have DR, we anticipated 2,160 people to have DR, of whom 216 to have STDR.

Considering that some patients would come from the same family, and some from the same area, we assumed that outcomes at the area level would have an allowed intra-centre correlation (ICC) coefficients of approximately up to 0.05 and to 0.10 for new- and known-diabetes respectively. At the area level, with approximately 100 cases per region, and a working ICC of 0.075, we expect a design effect of 8.5. This calculation has been based on conservative allowances and approximations, which allow for deviations in the actual intra-cluster correlation coefficients from those anticipated, or for variation in the actual number of cases across centres. This means that the effective sample size (were the sample to be free from clustering) is 25 STDR cases for covariates, which are constant at the region level, or highly correlated amongst families within the same area. Using the rule of 10 people per covariate in order to plan the number of possible covariates, this implies that it will be possible to include 10 to 20 covariates (216/10) at the participant level dependent on whether there is no, modest, or moderately high ICC in the covariates, and 1 to 2 covariates (25/2) either at the area/family level for a stable diagnostic STDR model. All models will include observations at the participant level in order to accommodate participant-level covariates and will accommodate clustering further by including two area contrast terms; these reflect whether a participant lives in the strata of regions that are urban, rural or a special population. Models will be from the 'marginal' class so that correlation can be accommodated while importantly retaining a participant-specific interpretation of resulting estimates. The study will continue to recruit to enable process evaluation and other sub-studies to be incorporated.

Health economics analysis plan

The health economics modelling will address the following three questions; (1) What is the cost-effectiveness of a new screening pathway for diabetes and pre-diabetes? The screening approaches will comprise: diabetes risk score followed by definitive laboratory tests; diagnostic model which the statistical modelling finds to be more accurate than diabetes risk score followed by definitive laboratory tests; RBG for all without diabetes risk score based pre-screen; HbA1c test with no pre-screen; no screening; (2) What is the cost-effectiveness of a new screening pathway for DR among people with diabetes? The screening approaches will comprise a new method which the statistical modelling finds to be accurate; retinal photographs only; no screening. (3) What is the cost-effectiveness of a new screening pathway for a range of other complications of diabetes among people with diabetes? The screening approaches will comprise a new method, which the statistical modelling finds to be accurate; a combination of HbA1c, lipids and urine tests and colour retinal images; no screening. In each case therefore one comparator will be a 'gold standard' (HbA1c test,

For peer review only

retinal photographs, combination of tests as above) and another will be no screening and no treatment until symptoms of DR, DKD or other complications of diabetes are experienced.

The modelling will draw on the following data sources: (1) The data collected through the house-to-house screening and associated retinal images, blood and urine tests on the rates of true and false positives and negatives, the characteristics of people with diabetes and its complications, and their quality of life. (2) Data collected through the study on the cost per person of this screening and its cost per person with diabetes, and the costs of clinic visits and treatments for DR. (3) Data and information from past studies on the incidence rates by age and gender of diabetes, DR and other complications of diabetes, transition rates between different stages of the disease, and disease-specific mortality rates. (4) Data from past studies on the costs of care for people with varying severities of DR and other complications of diabetes and on their quality of life. For those variables on which data cannot be collected in this study or obtained from past studies, expert views will be sought, and sensitivity analyses conducted.

The modelling will comprise development of Markov models to track people from age 40 onward (a) through incidence of diabetes, any DR, STDR, severe visual impairment/blindness and (b) through incidence of diabetes, mild complications other than DR, and severe complications other than DR. For each disease state the models will contain estimates of average annual costs of care and average EQ5D quality of life. The design of the models will be developed in the light of data availability.

The models will be used to estimate lifetime costs and quality of life (monetised quality adjusted life years, QALYs) from age 40 and older (a) where the planned screening approach (or approaches) is conducted and necessary treatment given shortly after screening; (b) where the 'gold standard' screening approach is conducted and necessary treatment given shortly after screening; and (c) where no screening is conducted and no treatment given until symptoms develop. The incremental cost-effectiveness of the screening in comparison with 'gold standard' screening will be estimated by comparing (a) and (b); and its incremental cost-effectiveness in comparison with no screening will be estimated by comparing (a) and (c). A wide range of sensitivity analysis will be conducted, and a variety of discount rates may be applied.

We will also evaluate and compare the cost-effectiveness of retinal photography for everyone with diabetes versus retinal photography only for people with diabetes with suspected high risk of DR, to be developed through the statistical modelling. We will develop a health economics plan after reviewing available data. As an example, Rachapelle et al used a WHO recommended approach for a cost-effectiveness threshold in their study of the cost-utility of telemedicine to screen for DR in India.[31] Under that approach, the interventions costing less than per capita Gross Domestic Product (GDP) per QALY were considered very cost-effective, interventions between 1 and 3 times GDP were considered cost-effective and interventions more than 3 times GDP were considered not cost-effective.

Process Evaluation

For peer review only

A detailed process evaluation plan will be developed to evaluate the holistic screening for all complications of diabetes including the teleophthalmology. For each quantitative outcome measure, we will systematically embed qualitative measures in each RE-AIM dimension (reach, efficacy, adoption, implementation, maintenance) to evaluate the implementation strategy of community screening with minimally invasive tests. [32,33]

Outcomes:

The primary outcome is the correlation of RBG levels and POC HbA1c levels. Secondary outcomes include the cut-off value of RBG to define pre-diabetes; diagnostic accuracy of risk stratification models for diabetes; prevalence and risk stratification for screening for diabetic retinopathy; risk model for those at risk of complications of diabetes; identification of cost-effective diagnostic model for diabetes, pre-diabetes and complications of diabetes and process evaluation of minimally invasive community screening for diabetes and its complications.

Patient and Public Involvement: No patient involved.

Ethics and dissemination

The Indian Council of Medical Research (ICMR) Health Ministry Screening Committee HMSC/2018-0494 dated 17/12/2018 and the Institutional Ethical Committees of all the participating Institutions have approved the study (Table 2). The main ethical issues in relation to this study are the identifications of people with risk factors for pre-diabetes, diabetes and its complications. However, the benefits of early diagnosis outweigh these risks. Participants who screen positive for any risk factors will be advised about referral to the local hospitals for treatment. Any breach of confidentiality will be minimised by anonymising participant identifiable information.

The results will be published in Open Access peer reviewed journals, presented at scientific meetings and shared with the funder, and specific communication will be organised to target health professionals, policy decision-makers, regulatory bodies and commercial bodies for development of better predictive devices. The anonymised study data will be analysed by the statistical team in the UK. Anonymised patient level data access will be made available to researchers from appropriate data archive for sharing purposes following publication of the study.

References

1. IDF Diabetes Atlas, 9th edition 2019 https://www.diabetesatlas.org/en/
2. Hostalek U. Global epidemiology of prediabetes - present and future perspectives. *Clin Diabetes Endocrinol* 2019; 5:5.
3. Bar-Tana J. Type 2 diabetes –unmet needs, unresolved pathogenesis, m-TORC1 centric paradigm *Rev Endocr Metab Disord* 2020;1-17.
4. Classification of diabetes. World Health Organisation. 2019 www.who.int/publications-detail/classification-of-diabetes-mellitus.
5. Standards of Medical Care in Diabetes. *Diabetes Care* 2019; 42(Suppl.1): S1-204.
6. Somannavar S, Ganesan A, Deepa M,et al. Random capillary blood glucose cut points for diabetes and pre-diabetes derived from community-based opportunistic screening in

For peer review only - http://bmjopen.bmj.com/site/about/guidelines.xhtml

India. *Diabetes Care* 2009;32(4):641–643.

7. Ziemer DC, Kolm P, Foster JK, Weintraub WS, Vaccarino V, Rhee MK, et al. Random plasma glucose in serendipitous screening for glucose intolerance: screening for impaired glucose tolerance study 2. *J Gen Intern Med.* 2008;23:528–5.
8. Rolka DB, Narayan KM, Thompson TJ, Goldman D, Lindenmayer J, Alich K, et al. Performance of recommended screening tests for undiagnosed diabetes and dysglycemia. *Diabetes Care.* 2001; 24:1899–3.
9. Susairaj P, Snehalatha C, Raghavan A, Nanditha A, Vinitha R, Satheesh K, Johnston DG, Wareham NJ, Ramachandran A. Cut-off Value of Random Blood Glucose among Asian Indians for Preliminary Screening of Persons with Prediabetes and Undetected Type 2 Diabetes Defined by the Glycosylated Haemoglobin Criteria*. J Diabetes Clin Res.* 2019;1(2):53-58.
10. Tahrani AA, Geen J, Hanna FW, Jones PW, Cassidy D, Bates D, Fryer AA. Predicting dysglycaemia in patients under investigation for acute coronary syndrome. *QJM.* 2011;104(3):231-6.
11. Badings EA, Dyal L, Schoterman L, Lok DJ, Stoel I, Gerding MN, Gerstein HC, Tijssen JG. Strategies to detect abnormal glucose metabolism in people at high risk of cardiovascular disease from the ORIGIN (Outcome Reduction with Initial Glargine Intervention) trial population. *J Diabetes.* 2011;3(3):232-7.
12. Ain Q, Latif A, Jaffar SR, Ijaz A. Evaluation of random plasma glucose for assessment of glycaemic control in type 2 diabetes mellitus. *J Pak Med Assoc.* 2017;67(9):1353-1356.
13. Gill GV, Hardy KJ, Patrick AW, Masterson A. Random blood glucose estimation in type 2 diabetes: does it reflect overall glycaemic control? Diabet Med. 1994;11(7):705-8.
14. Rasmussen JB, Nordin LS, Rasmussen NS, Thomsen JA, Street LA, Bygbjerg IC, Christensen DL. Random blood glucose may be used to assess long-term glycaemic control among patients with type 2 diabetes mellitus in a rural African clinical setting. *Trop Med Int Health.* 2014;19(12):1515-9.
15. Otieno FC, Ng'ang'a L, Kariuki M. Validity of random blood glucose as a predictor of the quality of glycaemic control by glycated haemoglobin in out-patient diabetic patients at Kenyatta National Hospital. East Afr Med J. 2002; 79(9):491-5.
16. Sicard DA1, Taylor JR Comparison of POC HbA1c test versus standardized laboratory testing. *Ann Pharmacother*2005;39(6):1024-8.
17. Barry E, Roberts S, Oke J, Vijayaraghavan S, Normansell R, Greenhalgh T. Efficacy and effectiveness of screen and treat policies in prevention of type 2 diabetes: systematic review and meta-analysis of screening tests and interventions. *BMJ.* 2017;356:i6538.
18. Mohan V, Anbalagan VP. Expanding role of the Madras Diabetes Research Foundation - Indian Diabetes Risk Score in clinical practice. *Indian J Endocrinol Metab*2013;17(1):31-6.
19. Joseph P, Yusuf S, Lee SF, et al. PURE INVESTIGATORS. Prognostic validation of a non-laboratory and a laboratory based cardiovascular disease risk score in multiple regions of the world. *Heart*2018:104(7):581-87.

For peer review only

20. Scanlon PH, Aldington SJ, Leal J, et al. Development of a cost-effectiveness model for optimisation of the screening interval in diabetic retinopathy screening. *Health Technol Assess*2015;19(74):1-116.
21. Natarajan S, Jain A, Krishnan R, et al. Diagnostic Accuracy of Community-Based Diabetic Retinopathy Screening With an Offline Artificial Intelligence System on a Smartphone. *JAMA Ophthalmol*2019:137(10);1182-88.
22. Rajalakshmi R, Arulmalar S, Usha M et al., Validation of Smartphone Based Retinal Photography for Diabetic Retinopathy Screening. *PLoS One*2015:10(9):e0138285.
23. Wilkinson CP, Ferris FL 3rd, Klein RE, et al. Global Diabetic Retinopathy Project Group. Proposed international clinical diabetic retinopathy and diabetic macular edema disease severity scales. *Ophthalmology*2003;110(9):1677-82.
24. Misajon R, Hawthorne G, Richardson J, et al. Vision and quality of life: the development of a utility measure. *Invest Ophthalmol Vis Sci*2005;46(11):4007-15.
25. Peacock S, Misajon R, Iezzi A, et al. Vision and quality of life:development of methods for the VisQoL vision-related utility instrument. *Ophthalmic Epidemiol*2008;15(4):218-23.
26. Janssen MF, Birnie E, Bonsel GJ. Quantification of the level descriptors for the standard EQ-5D three-level system and a five-level version according to two methods. *Qual Life Res*2008;17(3):463-73.
27. The WHO STEPwise approach to surveillance of noncommunicable diseases (STEPS). Noncommunicable Diseases and Mental Health. World Health Organization. 20 Avenue Appia, 1211 Geneva 27, Switzerland. http://www.who.int/ncd_surveillance
28. Royston P, Moons KGM, Altman DG, et al. Prognosis and prognostic research: Developing a prognostic model. *BMJ*2009;338:b604.
29. Sauerbrei W, Royston P. Building multivariable prognostic and diagnostic models: transformation of the predictors by using fractional polynomials. *J R Stat Soc Series A*1999;162:71-94
30. Altman DG, Vergouwe Y, Royston P, et al. Prognosis and prognostic research:validating a prognostic model. *BMJ*2009;338:b605
31. Rachapelle S, Legood R, Alavi Y, et al. The cost-utility of telemedicine to screen for diabetic retinopathy in India. *Ophthalmology*2013;120(3):566-73.
32. Moore GF, Audrey S, Barker M, et al., Process evaluation of complex interventions: Medical Research Council guidance. *BMJ*2015;350:h1258.
33. Forman J, Heisler M, Damschroder LJ, et al. Development and application of the RE-AIM QuEST mixed methods framework for program evaluation. *Prev Med Rep*2017; 6:322-28.

Legends for figures:

Figure 1: Map of India with 20 centres marked.

Figure 2: Study flow diagram

Author Statement

For peer review only

SMART India writing group: Sobha Sivaprasad (S.S), Rajiv Raman (R.R SN), Ramachandran Rajalakshmi (R.R MDRF), Viswanathan Mohan (V.M), Deepa Mohan (D.M), Taraprasad Das (T.D), Kim Ramasamy (K.R), Toby Prevost (T.P), Raphael Wittenberg (R.W), Gopal Netuveli (G.N), Gopal Lingam (G.L), Wasim Hanif (W.H), Dolores Conroy (D.C), Jayashree Ramu (J.R), Janani Surya (J.S) and Radha Ramakrishnan (R.R UCL).

Conceptualisation, S.S, R.R (SN), R.R (MDRF), V.M, K.R, D.M, J.R, T.P, G.L, T.D, G.N, R.W; methodology, S.S, T.D, G.N, R.W, T.P, J.R, D.M, R.R (UCL); formal analysis, S.S, G.N, R.W, T.P, R.R (UCL); writing—original draft preparation, S.S, D.C, R.R (SN), R.R (MDRF), T.D, G.N, R.W, J.S, W.H, J.R, T.P; writing—review and editing, S.S, R.R (SN), R.R (MDRF), V.M, K.R, G.L, T.D, G.N, R.W, J.R, T.P, W.H, D.M, D.C, R.R (UCL); funding acquisition, S.S, R.R (SN), R.R (MDRF), V.M, K.R, T.D, G.N, R.W. on behalf of the SMART India Collaborators in Table 2.

Table 2.: SMART-India Collaborators

Site No.	Name of Principal Investigator	Hospital Name	Ethics approval and date
1	Dr Pramod Bhende<br>Dr Rajiv Raman	Sankara Nethralaya, Chennai, Tamil Nadu	Vison Research Foundation Institutional Review Board<br>Study code:VRF/674A-2018-P<br>Date of approval: 22nd March 2018
2	Dr Ramachandran Rajalakshmi<br>Dr Viswanathan Mohan	Dr Mohan's Diabetes Specialities Centre, Chennai, Tamil Nadu	Madras Diabetes Research Foundation Institutional Ethics Committee<br>Date of approval: 6th March 2018. Reference number MDRF/NCT/02–01/2018
3	Dr Kim Ramasamy	Aravind Eye Hospital, Madurai, Tamil Nadu	Aravind Medical Research Foundation Institutional Ethics Committee Reg No: ECR/182/Inst/TN/2013/RR-19<br>IRB2018010BAS<br>Date of approval:21st Apr 2018
4	Dr Taraprasad Das<br>Dr Padmaja K Rani	LV Prasad Eye Institute, Hyderabad, Telangana	LV Prasad Eye Institute Ethics Committee Ref: LEC07-18-096<br>Date of approval:19th July 2018
5	Dr Rupak Roy<br>Dr Supita Das	Sankara Nethralaya, Kolkata	Vison Research Foundation Institutional Review Board<br>Study code:VRF/674A-2018-P<br>Date of approval: 22nd March 2018
6	Dr Deepa Mohan	Dr Mohan's Diabetes Specialities Centre, Mysuru, Karnataka	Madras Diabetes Research Foundation Institutional Ethics Committee

			Date of approval: 6th March 2018. Reference number MDRF/NCT/02–01/2018
7	Dr V Narendran<br>Dr George Manayath	Aravind Eye Hospital, Coimbatore, Tamil Nadu	Aravind Medical Research Foundation Institutional Ethics Committee<br>ECR/182/Inst/TN/2013<br>IRB2018010BAS<br>Date of approval: 18th Aug 2018
8	Dr Giridhar Anantharaman<br>Dr Mahesh Gopalakrishnan	Giridhar Eye Institute, Cochin, Kerala	Giridhar Eye Institute Ethics Committee<br>IEC protocol no:36/2018<br>Date of approval: 13th June 2018
9	Dr Sundaram Natarajan<br>Dr Radhika Krishnan	Aditya Jyot Hospital, Mumbai, Maharashtra	Aditya Jyot Eye Hospital Ethics Committee<br>Date of approval: 30th Aug 2018
10	Dr Sheena Liz Mani	Dr Tony Fernandez Eye Hospital, Aluva, Kerala	Dr Tony Fernandez Eye Hospital Ethics Committee<br>Date of approval: 21st June 2018
11	Dr Manisha Agarwal	Dr Shroff's Charity Eye Hospital, New Delhi	Dr Shroff's Charity Eye Hospital Ethics Committee<br>Date of approval: 29th Jan 2018
12	Dr Tapas Padhi<br>Dr Umesh Behera	LV Prasad Eye Institute, Bhubaneshwar, Odisha	LVPEI Bhubaneswar Ethics Committee<br>Date of approval 10th Oct 2018
13	Dr Harsha Bhattacharjee<br>Dr Manabjyoti Barman	Sri Sankaradeva Nethralaya, Guwahati, Assam	Sri Sankaradeva Nethralaya Institutional Ethics Committee<br>Ref number: SSN/IEC/OCTOBER/2018/09<br>Date of approval:8th Oct 2018
14	Dr Gajendra Chawla	Vision Academy- The Socio Medical Society, *Bhopal, Madhya Pradesh*	Vision Research Foundation Chennai Institution Review Committee<br>Approval number - 674A-2018-P<br>Date of approval 22nd March 2018
15	Dr Alok Sen	Sadguru Netra Chikitsalaya, Chitrakoot, Madhya Pradesh	Vision Research Foundation, Chennai Institutional Review Committee<br>Approval number - 674A-2018-P<br>Date of approval 22nd March 2018
16	Dr Moneesh Saxena	Aurobindo Nethralaya, Raipur, Chhattisgarh	Shri Aurobindo Medical Research Centre Institutional Review Board<br>Date of approval:22nd June 2018

For peer review only

17	Dr Asim K Sil<br>Dr Subhratanu Chakabarty	Netra Niramay Niketan, Haldia, West Bengal	Vivekendra Mission Asram Netra Niramay Niketan Institutional Review Board<br>Date of approval 4th September 2018
18	Dr Thomas Cherian<br>Dr Reesha KR	Little Flower Hospital & Research Center, Angamaly, Kerala	Little Flower Hospital and Research Centre Ethics Committee<br>Date of approval:4th June 2018
19	Dr Rushikesh Naigaonkar<br>Dr Abishek Desai	Ganapathy Nethralaya, Jalna, Maharashtra	Shri Ganapati Netralaya Institutional Ethics Committee<br>Date of approval: 28th July 2018
20	Dr Col Madan Deshpande<br>Dr Sucheta Kulkarni	HV Desai Hospital, Pune, Maharashtra	PBMA's H. V. Desai Eye Hospital Institutional Review Committee. HVD/ EC/ 17/ 2018<br>Date of approval:21st June 2018

Acknowledgements: The authors would like to thank all the SMART-India collaborators including field workers, each centre staff, reading centre staff, Jitendra Pal Thethi for the study database; and the study participants.

Funding statement: This work was supported by Global Challenges Research Fund and UK Research and Innovation through the Medical Research Council grant number MR/P027881/1. This funding source had no role in the design of this study and will not have any role during its execution, analyses,interpretation of the data, or decision to submit results.

Data Sharing Agreement: The technical appendix, statistical code and deidentified dataset will be made available on an appropriate data archive for sharing purposes following publication of the study.

Competing interests statement: None declared

For peer review only

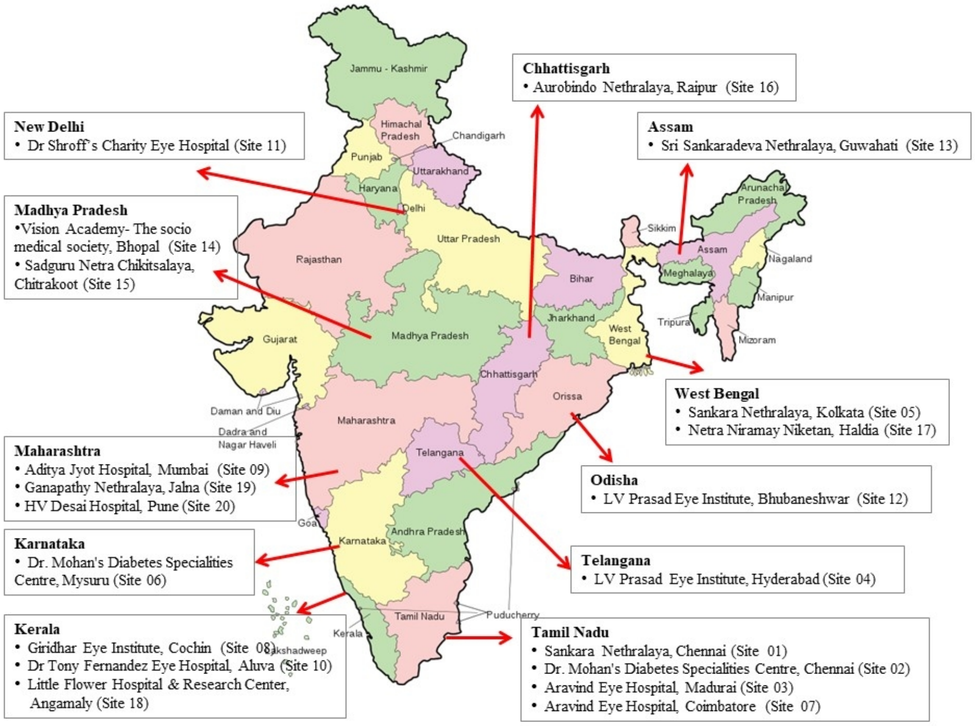

Figure 1: Map of India with 20 centres marked

81x60mm (300 x 300 DPI)

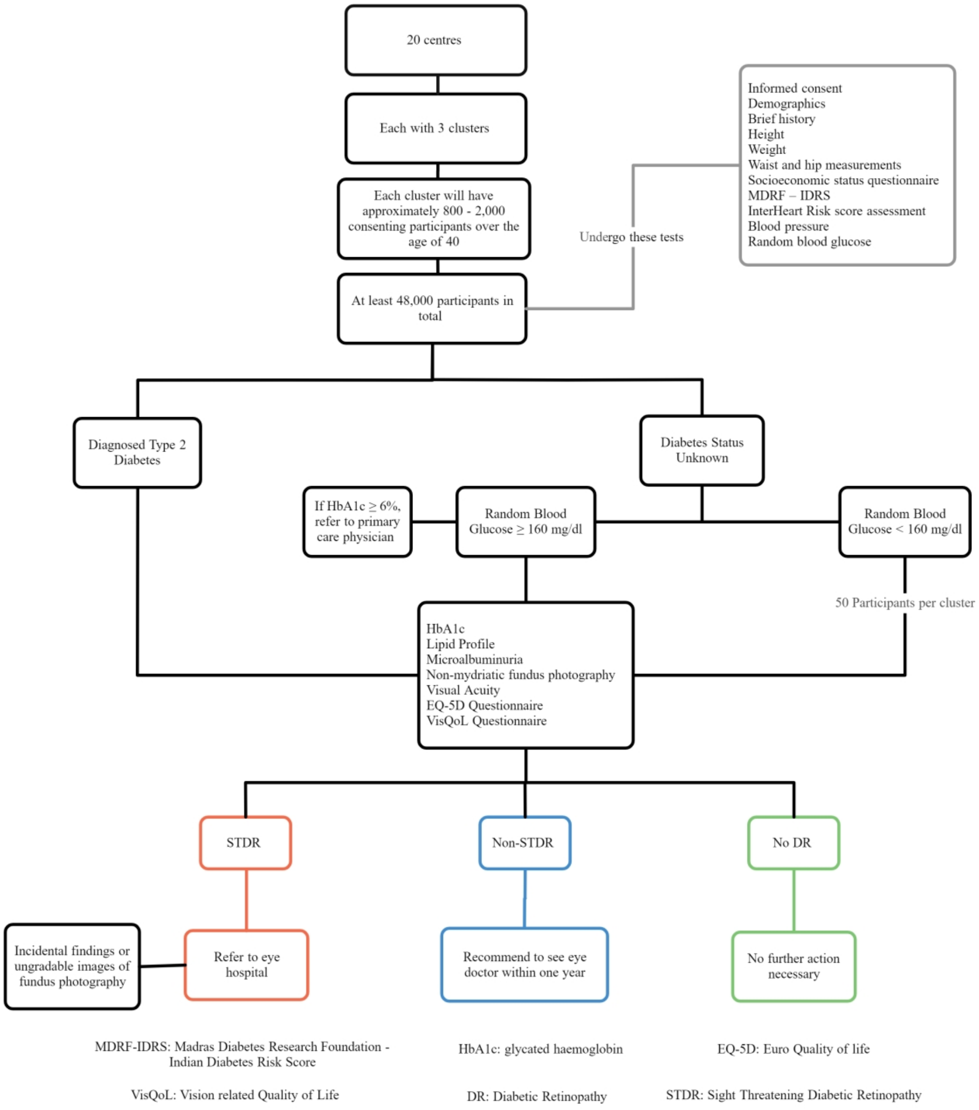

Figure 2: Study flow diagram

INFORMED CONSENT FORM
FOR PARTICIPATION IN THE SMART INDIA STUDY

India has the second largest number of people with diabetes in the world and the number is increasing every year. It is well known that people with diabetes are at a higher risk of getting eye problems, heart attack/ stroke, or kidney disease. Some people may have altered blood sugar levels before they actually develop diabetes. This is an All India study which is being done to find out the burden of pre-diabetes and diabetes and the complications due to diabetes, especially the eye complication of diabetes called retinopathy. For this purpose you will be asked some questions which will be recorded in a questionnaire. Blood pressure and a few anthropometric measurements will be taken. All people will then have a finger prick blood test done and photo of the back of the eye (retina) taken using a simple retinal camera. Some additional blood tests and urine test will be done for a subset of people. It is possible that this study could determine that you have diabetes and / or its associated disorders. If so, you will benefit from this information as you can seek early treatment for these disorders. The information you provide in the questionnaire, results of your blood tests and retinal photography will be kept confidential.

**Patient identification number for this study**	
**Title of the project**	**SMART INDIA study**(Statistical **M**odelling and **R**isk **A**ssessment of **T**ype 2 diabetes complications in India)
**Name of Principal Investigator (s)**	

The contents of the patient information sheet that has been provided have been read carefully by me/explained in detail to me, in a language that I comprehend, and I have fully understood the contents.
I confirm that I have had the opportunity to ask questions. The nature and purpose of the study and its potential risks / benefits and expected duration of the study, and other relevant details have been explained to me in detail. I understand that my participation in this study is voluntary and that I am free to withdraw at any time, without giving any reason.
I understand that the information collected about me from participation in this study and sections of any of the results may be looked at by responsible individuals involved in this research project either in India or outside India. Anonymised data and retinal images may be shared with other researchers.
I agree to take part in the above study.

-- Date:
(Signature/Left Thumb impression of participant)
Place:

Name of the Participant: _ _ _ _ _ _ _ _ _ _ _ _ _ _ _ _ _ _

Son/Daughter/spouse of: _ _ _ _ _ _ _ _ _ _ _ _ _ _ _ _ _ _

Complete postal address: _ _ _ _ _ _ _ _ _ _ _ _ _ _ _ _ _ _

1) Witness

-------------------------------------- Date:
(Signature)

Name

Address:

For peer review only - http://bmjopen.bmj.com/site/about/guidelines.xhtml
For peer review only

SMART India	Participant ID:							Participant Initials		
	Date of Consent:							Year of birth:		

SMART India study Questionnaire

* All questionnaires must be interviewer administered

S.No	Check List	YES	NO
1	Household details		
2	Demographic data and Anthropometric measurements (Main survey)		
3	Diabetes Information		
4	EQ5D questionnaire		
5	Vision Quality of Life questionnaire (VisQoL)		
6	Cost data/Expenses form		
7	Fundus Image		

Person administering the questionnaire	
**Signature**	
**Name**	
**Participant who is administered**	
**Signature**	
**Name**	

SMART India	Participant ID:							Participant Initials		
	Date of Consent:							Year of birth:		

PART 1 – House hold Details- House Survey Record

1	Centre		
2	Region Type	1	Urban
		2	Rural
		3	Special
3	Address		
4	Phone / Mobile Number:		
5	City		
6	Pin		
7	Household Status	1	No one available in this household
		2	Household not willing to participate
		3	Available

*If **1** or **2** skip question No.**8***

8	If available, number of people in house above 40 years	
9	Enter details of people in the house hold	

For peer review only

SMART India	Participant ID:							Participant Initials		
	Date of Consent:							Year of birth:		

9.1. Person-1			
a	Participation	1	Willing to take part
		2	Type 1 diabetic - exclusion
		3	Gestational diabetes - exclusion
		4	Other exclusion
	If 4, Reason		

*If **3** skip "**b**", if **1 or 2** skip "**c and d**"*

b	Gender	1	Male
		2	Female
		3	Other (do not want to disclose, transgender, etc.)

c	Name of the person 1<br>*Example: Ajith **K**umar*	

d	Initials of the person 1<br>*Example: **AK***	

9.2 Person-2			
a	Participation	1	Willing to take part
		2	Type 1 diabetic - exclusion
		3	Gestational diabetes - exclusion
		4	Other exclusion
	If 4, Reason		

*If **3** skip "**b**", if **1 or 2** skip "**c and d**"*

b	Gender	1	Male
		2	Female
		3	Other (do not want to disclose, transgender, etc.)

c	Name of the person 2	

d	Initials of the person 2	

For peer review only - http://bmjopen.bmj.com/site/about/guidelines.xhtml

SMART India	Participant ID:							Participant Initials		
	Date of Consent:							Year of birth:		

9.3. Person-3			
a	Participation	1	Willing to take part
		2	Type 1 diabetic - exclusion
		3	Gestational diabetes - exclusion
		4	Other exclusion
	If 4, Reason		

*If **3** skip "**b**", if **1 or 2** skip "**c and d**"*

b	Gender	1	Male
		2	Female
		3	Other (do not want to disclose, transgender, etc.)

c	Name of the person 3	

d	Initials of the person 3	

9.4. Person-4			
a	Participation	1	Willing to take part
		2	Type 1 diabetic - exclusion
		3	Gestational diabetes - exclusion
		4	Other exclusion
	If 4, Reason		

*If **3** skip "**b**", if **1 or 2** skip "**c and d**"*

b	Gender	1	Male
		2	Female
		3	Other (do not want to disclose, transgender, etc.)

c	Name of the person 4	

d	Initials of the person 4	

For peer review only - http://bmjopen.bmj.com/site/about/guidelines.xhtml

SMART India	Participant ID:							Participant Initials				
	Date of Consent:							Year of birth:				

9.5. Person -5			
a	Participation	1	Willing to take part
		2	Type 1 diabetic - exclusion
		3	Gestational diabetes - exclusion
		4	Other exclusion
	If 4, Reason		

*If **3** skip "**b**", if **1 or 2** skip "**c** and **d**"*

b	Gender	1	Male
		2	Female
		3	Other (do not want to disclose, transgender, etc.)

c	Name of the person 5	

d	Initials of the person 5	

9.6. Person - 6			
a	Participation	1	Willing to take part
		2	Type 1 diabetic - exclusion
		3	Gestational diabetes - exclusion
		4	Other exclusion
	If 4, Reason		

*If **3** skip "**b**", if **1 or 2** skip "**c** and **d**"*

b	Gender	1	Male
		2	Female
		3	Other (do not want to disclose, transgender, etc.)

c	Name of the person 6	

d	Initials of the person 6	

For peer review only - http://bmjopen.bmj.com/site/about/guidelines.xhtml

SMART India	Participant ID:							Participant Initials		
	Date of Consent:							Year of birth:		

PART 2 – Demographic data and Anthropometric measurements

Instructions:

READ CATEGORIES for all questions. CIRCLE ONE

1	Participant ID:	

2	Date of Consent:	/ /

3	Year of Birth:	

Note: *Choose between 1920 to 1978*

4	Gender:	1	Male
		2	Female
		3	Other (do not want to disclose, transgender, etc.)

5	Highest level of Education: (Select Education Level)	1	None
		2	Primary
		3	Secondary
		4	Graduate
		5	Postgraduate or higher
		6	Not classified

6	Occupation: (select occupation)	1	Not working due to health reasons
		2	Not working due to vision reasons
		3	Housewife
		4	Unemployed
		5	Retired
		6	Unskilled worker
		7	Skilled worker
		8	Professional
		9	Self Employed

For peer review only - http://bmjopen.bmj.com/site/about/guidelines.xhtml

SMART India	Participant ID:							Participant Initials		
	Date of Consent:							Year of birth:		

7	Average Monthly Individual Income (Rs.)	1	Do not want to disclose
		2	

Enter valid Income (0-10000000)

8	Smoking Status	1	Non-smoker
		2	Former smoker
		3	Smoker
			*If **1** or **2** Go to **9***
8a	No of cigarettes per day:		

Please enter valid value (1-99)

9	Second hand smoke exposure for one or more hours per week:	1	No
		2	Yes

10	Physical Exercise (Select from list)	1	Sedentary
		2	Mild exercise
		3	Moderate exercise
		4	Vigorous or strenuous exercise

11	Several periods of stress or permanent stress in the last year (select Yes or No)	1	No
		2	Yes

12	In the last year, was there a time when you felt sad, blue or depressed for two weeks or more in a row (*select Yes or No*)	1	No
		2	Yes

13	Diet: (Select all that applies)<br>At least one option should be selected.	1	Salty food or snacks one or more times a day
		2	Deep fried foods or snacks or fast foods 3 or more times per week
		3	Eat fruit less than once per day
		4	Eat vegetables less than once per day
		5	Eat meat and / or poultry 2 or more times daily
		6	None of the above

For peer review only - http://bmjopen.bmj.com/site/about/guidelines.xhtml

SMART India	Participant ID:							Participant Initials		
	Date of Consent:							Year of birth:		

14	Diagnosed diabetes Type 2 (Only Type 2 eligible)	1	Don't know
		2	No
		3	Yes

*If "**Don't Know or NO** " Go to **15***

14a	Duration of diabetes Type 2 since diagnosis. (enter duration in years and 0 – 11 months)	Years:	Months:

14b	Treatment of Diabetes Mellitus:	1	None / Diet controlled
		2	Oral hypoglycaemic agents only
		3	Insulin only
		4	Both insulin and oral hypoglycaemic agent

14c	Complications of diabetes mellitus (Select all that applies)	1	None
		2	Chronic kidney disease
		3	Peripheral neuropathy (diabetic foot)
		4	Diabetic retinopathy

14d	Are you aware that diabetes can cause blindness?	1	No
		2	Yes

15	Cardiovascular disease (Select all that applies)	1	None
		2	Hypertension
		3	Myocardial infarction
		4	Heart failure
		5	Stroke
		6	Transient ischaemic attack

16	Medical History - any other history not covered above	

17	Ocular history (Select all that applies): *At least one option should be selected*	1	None
		2	Cataract present
		3	Cataract surgery done in at least 1 eye
		4	Glaucoma
		5	AMD (age related macular degeneration)

For peer review only - http://bmjopen.bmj.com/site/about/guidelines.xhtml

**SMART India**	**Participant ID:**							**Participant Initials**		
	**Date of Consent:**							**Year of birth:**		

17a	Other Ocular History - *any other history not covered before*	

18	Parental history of diabetes	1	Both non-diabetic
		2	Either parents diabetic
		3	Both parents diabetic

19	Parental history of heart attack	1	No
		2	Yes

20	Height (cms)<br>*Enter Valid Height in cms (100-230)*	

21	Weight (kgs)<br>*Enter valid weight in kgs (30-300)*	

22	Waist circumference (cms)<br>*Enter valid value in cms (20-300)*	

23	Hip circumference (cms)<br>*Enter valid value in cms (20-300)*	

24	Systolic Blood pressure (mm Hg)<br>*Enter valid value (30 - 250) and above Diastolic*	

25	Diastolic Blood pressure (mm Hg)<br>*Enter valid value (30 - 250)*	

For peer review only

SMART India	Participant ID:							Participant Initials		
	Date of Consent:							Year of birth:		

Part 3- Diabetes Information

1	Participant ID:		

2	Diabetes:	1	No/Don't know
		2	Yes

3	Random Blood sugar (mg/dl):<br>*Enter valid value (50 - 500)*	

If patient is known diabetic, then whatever the value of RBS, all tests must be carried out.
If diabetes 'No / Unknown – RBS < 110 – (End of Survey)
RBS between 110 and 160 – Answer 'Carry out all tests ?' Yes – No

3a	Carry Out All Tests?	1	No
		2	Yes

4	HbA1c (%):<br>*Enter valid value (4-13)*	

5	Microalbuminuria:	1	No
		2	Yes
		3	Urine sample not available

6	Total Cholesterol – mg/dL<br>*Enter Valid value (100-400)*	

7	HDL Cholesterol – mg/dL<br>*Enter Valid value (20-120)*	

8	Total Triglycerides – mg/dL<br>*Enter Valid value (50-500)*	

9	LDL Cholesterol – mg/dL<br>*Enter Valid value (0-450)*	

10	Total Cholesterol / HDL Ratio<br>*Enter Valid value (1-33.3)*	

11	Non-HDL Cholesterol – mg/dL<br>*Enter Valid value (0-450)*	

For peer review only - http://bmjopen.bmj.com/site/about/guidelines.xhtml

**SMART India**	**Participant ID:**							**Participant Initials**			
	**Date of Consent:**							**Year of birth:**			

12	Distance Vision in right eye (with glasses if available) *Select from list*	1	0.0
		2	0.1
		3	0.2
		4	0.3
		5	0.4
		6	0.5
		7	0.6
		8	0.7
		9	0.8
		10	0.9
		11	1.0
		12	1.1
		13	1.2
		14	Worse than or equal to 1.3

13	Distance Vision in left eye (with glasses if available) *Select from list*	1	0.0
		2	0.1
		3	0.2
		4	0.3
		5	0.4
		6	0.5
		7	0.6
		8	0.7
		9	0.8
		10	0.9
		11	1.0
		12	1.1
		13	1.2
		14	Worse than or equal to 1.3

14	Were the fundus photographs taken? *Please enter the Participant ID in fundus system*	1	Yes
		2	Not obtainable
NOTE: If 2 :Please capture the participants front of the eye and upload it in the upload page, if the image is not obtainable			

For peer review only

SMART India	Participant ID:							Participant Initials		
	Date of Consent:							Year of birth:		

PART 4 – Eq5d questionnaire

By placing a tick in one box in each group below, please indicate which statements best describe your own health state **TODAY**

1	Mobility	1	I have no problems in walking about
		2	I have slight problems in walking about
		3	I have moderate problems in walking about
		4	I have severe problems in walking about
		5	I am unable to walk about

2	Self-care	1	I have no problems washing or dressing myself
		2	I have mild problems washing or dressing myself
		3	I have moderate problems washing or dressing myself
		4	I have severe problems washing or dressing myself
		5	I am unable to wash or dress myself

3	Usual Activities (*e.g. work, study, housework, family or leisure activities)*	1	I have no problems with performing my usual activities
		2	I have mild problems with performing my usual activities
		3	I have moderate problems with performing my usual activities
		4	I have severe problems with performing my usual activities
		5	I am unable to perform my usual activities

4	Pain / Discomfort	1	I have no pain or discomfort
		2	I have mild pain or discomfort
		3	I have moderate pain or discomfort
		4	I have severe pain or discomfort
		5	I have extreme pain or discomfort

5	Anxiety / Depression	1	I am not anxious or depressed
		2	I am mildly anxious or depressed
		3	I am moderately anxious or depressed
		4	I am severely anxious or depressed
		5	I am extremely anxious or depressed

6	Vision *(using glasses or contact lenses if needed)*	1	I have no problems seeing
		2	I have slight problems seeing
		3	I have some problems seeing
		4	I have severe problems seeing
		5	I am unable to see

For peer review only

SMART India	Participant ID:		Participant Initials	
	Date of Consent:		Year of birth:	

7	How good or bad your health state, is imagined in a scale 0 to 100. *The best state you can imagine is written as 100 and the worst state you can imagine is written as 0.*	

Enter value between (0 – 100)

8	Life satisfaction: All things considered, how satisfied are you with your life as a whole these days in 1 to 10 scale? *Please mark on the scale where 1 is dissatisfied* and 10 is satisfied.	

Enter value between(0 – 10)

Part 5 - Vision quality of life questionnaire

1	Does my vision make it likely I will injure myself (i.e., when moving around the house, yard, neighbourhood, or workplace)?	1	It is most unlikely I will injure myself because of my vision
		2	There is a small chance
		3	There is a good chance
		4	It is very likely
		5	Almost certainly my vision will cause me to injure myself

2	Does my vision make it difficult to cope with the demands in my life?<br><br>My vision:	1	Has no effect on my ability to cope with the demands in my life
		2	Does not make it difficult at all to cope with the demands in my life
		3	Makes it a little difficult to cope
		4	Makes it moderately difficult to cope
		5	Makes it very difficult to cope
		6	Makes me unable to cope at all

3	Does my vision affect my ability to have friendships?<br><br>My vision:	1	Makes having friendships easier
		2	Has no effect on my friendships
		3	Makes friendships more difficult
		4	Makes friendships a lot more difficult

For peer review only - http://bmjopen.bmj.com/site/about/guidelines.xhtml

SMART India	Participant ID:							Participant Initials		
	Date of Consent:							Year of birth:		

		5	Makes friendships extremely difficult
		6	Makes me unable to have friendships
		7	Not applicable; I have no friendships

4	Do I have difficulty organizing any assistance I may need?	1	I have no difficulty organizing any assistance I may need
		2	I have a little difficulty organizing assistance
		3	I have moderate difficulty organizing assistance
		4	I have a lot of difficulty organizing assistance
		5	I am unable to organize assistance at all
		6	Not applicable; I never need to organize assistance

5	Does my vision make it difficult to fulfil the roles I would like to fulfil in life (e.g., family roles, work roles, community roles)?<br><br>My vision:	1	Has no effect on my ability to fulfil these roles
		2	Does not make it difficult to fulfil these roles
		3	Makes it a little difficult to fulfil these roles
		4	Makes it moderately difficult to fulfil these roles
		5	Makes it very difficult to fulfil these roles
		6	Means I am unable to fulfil these roles

6	Does my vision affect my confidence to join in everyday activities?<br><br>My vision:	1	Makes me more confident to join in everyday activities
		2	Has no effect on my confidence to join in everyday activities
		3	Makes me feel a little less confident
		4	Makes me feel moderately less confident
		5	Makes me feel a lot less confident
		6	Makes me not confident at all

For peer review only - http://bmjopen.bmj.com/site/about/guidelines.xhtml

SMART India	Participant ID:							Participant Initials		
	Date of Consent:							Year of birth:		

Part 6 - Expense form

Instructions: Fill the expenses form only for those who are diabetic (if PART 2: 14 = "YES")

1a	Have you seen an eye doctor for diabetic eye disease in the last 3 years?	1	No
		2	Yes

*If '**No**' skip all question in expense form (skip **1b to 4**)*

1b	Have you been diagnosed with diabetic eye disease?	1	No
		2	Yes

*If '**No**' skip to 2a question*

1c	Have you received any treatment for diabetic eye disease in the last one year? (*Select all that applies*)	1	No Treatment
		2	Laser (Macular / PRP)
		3	Injection into the Eye (Anti-VEGF / Steroids)
		4	Surgery (Vitrectomy)

At least one option should be selected

1d	How was your vision before treatment?	1	I had no problems seeing
		2	I had slight problems seeing
		3	I had some problems seeing
		4	I had severe problems seeing
		5	I was unable to see

1e	Have you noticed an improvement in your vision following treatment?	1	No change
		2	Improved
		3	Worsened

2a	What were the total costs in last one year for treatment of diabetic eye disease (treatment / consultation / surgery)	Rs.	

Enter valid number (>= 0 and less than 999999)

2b	If you received any treatment including consultations in the last one year for diabetic eye disease, was the treatment	1	Free
		2	Concessional Cost
		3	Paid In Full

3	What were the travel costs for you and your carer (family member) in the last one year to go to the eye doctors, eye hospitals etc. for treatment of diabetic eye disease	Rs.	

For peer review only - http://bmjopen.bmj.com/site/about/guidelines.xhtml

SMART India	Participant ID:							Participant Initials		
	Date of Consent:							Year of birth:		

Enter valid number (>= 0 and less than 999999)

4	Did you have to take time off work due to diabetic eye disease treatment in the last one year?	1	No
		2	Yes

5a	Do you think you have visual impairment?	1	No
		2	Yes
5b	Does your visual impairment affect your ability to work?	1	No
		2	Yes

6	Did you receive any inpatient treatment for kidney disease in the last one year?	1	No
		2	Yes

7	Did you receive any inpatient treatment for heart condition or stroke in the last one year?	1	No
		2	Yes

8	Did you receive any treatment for diabetic foot disease (Ulcer / Gangrene/ Amputation) in the last one year?	1	No
		2	Yes

9 a	What were the costs in last one year for treatment of diabetes or its complications (heart conditions, kidney problems, feet problems etc) other than diabetic eye disease		
	Break Up	Medications	Rs.
		Investigations	Rs.
		Consultations	Rs.
		Hospitalization	Rs.
		Sum	Rs.
	Or		
	Total	Rs.	

9 b	If you received any treatment in the last one year for diabetes or its complications (heart conditions, kidney problems, feet problems etc), was the treatment…	1	Free
		2	Concessional Cost
		3	Pain In Full

10	What were the travel costs for you and your carer (family member) in the last one year to go to the doctors, hospitals etc for treatment of diabetes or its complications (exclude diabetic eye disease costs)	Rs.	

Enter valid number (>= 0 and less than 999999)

SMART India	Participant ID:							Participant Initials		
	Date of Consent:							Year of birth:		

11	Did you have to take time off work due to diabetes or its complications treatment (other than diabetic eye disease) in the last one year?	1	No
		2	Yes

PART 7 - Fundus Image

Instruction:

Please enter the Participant ID in fundus system. Capture Macula centered and Disc centered images and upload minimum 4 images of good quality to the database.

Please capture the participant's front of the eye and upload it in the upload page, **if the image is not obtainable.**

Please write the Fundus cam image ID if unable to transfer the image to database

**Image No**	**Image ID**	
	**OD**	**OS**
**1**		
**2**		
**3**		
**4**		
**5**		
**6**		
**7**		
**8**		

For peer review only

STANDARD PROTOCOL ITEMS: RECOMMENDATIONS FOR INTERVENTIONAL TRIALS

SPIRIT 2013 Checklist: Recommended items to address in a clinical trial protocol and related documents*

Section/item	Item No	Description	Addressed on page number
**Administrative information**			
Title	1	Descriptive title identifying the study design, population, interventions, and, if applicable, trial acronym	1
Trial registration	2a	Trial identifier and registry name. If not yet registered, name of intended registry	2
	2b	All items from the World Health Organization Trial Registration Data Set	________
Protocol version	3	Date and version identifier	2
Funding	4	Sources and types of financial, material, and other support	18
Roles and responsibilities	5a	Names, affiliations, and roles of protocol contributors	16
	5b	Name and contact information for the trial sponsor	2
	5c	Role of study sponsor and funders, if any, in study design; collection, management, analysis, and interpretation of data; writing of the report; and the decision to submit the report for publication, including whether they will have ultimate authority over any of these activities	18
	5d	Composition, roles, and responsibilities of the coordinating centre, steering committee, endpoint adjudication committee, data management team, and other individuals or groups overseeing the trial, if applicable (see Item 21a for data monitoring committee)	9

For peer review only

**Introduction**			
Background and rationale	6a	Description of research question and justification for undertaking the trial, including summary of relevant studies (published and unpublished) examining benefits and harms for each intervention	4
	6b	Explanation for choice of comparators	NA
Objectives	7	Specific objectives or hypotheses	5
Trial design	8	Description of trial design including type of trial (eg, parallel group, crossover, factorial, single group), allocation ratio, and framework (eg, superiority, equivalence, noninferiority, exploratory)	6
**Methods: Participants, interventions, and outcomes**			
Study setting	9	Description of study settings (eg, community clinic, academic hospital) and list of countries where data will be collected. Reference to where list of study sites can be obtained	6, 16
Eligibility criteria	10	Inclusion and exclusion criteria for participants. If applicable, eligibility criteria for study centres and individuals who will perform the interventions (eg, surgeons, psychotherapists)	7
Interventions	11a	Interventions for each group with sufficient detail to allow replication, including how and when they will be administered	NA
	11b	Criteria for discontinuing or modifying allocated interventions for a given trial participant (eg, drug dose change in response to harms, participant request, or improving/worsening disease)	NA
	11c	Strategies to improve adherence to intervention protocols, and any procedures for monitoring adherence (eg, drug tablet return, laboratory tests)	NA
	11d	Relevant concomitant care and interventions that are permitted or prohibited during the trial	NA
Outcomes	12	Primary, secondary, and other outcomes, including the specific measurement variable (eg, systolic blood pressure), analysis metric (eg, change from baseline, final value, time to event), method of aggregation (eg, median, proportion), and time point for each outcome. Explanation of the clinical relevance of chosen efficacy and harm outcomes is strongly recommended	13
Participant timeline	13	Time schedule of enrolment, interventions (including any run-ins and washouts), assessments, and visits for participants. A schematic diagram is highly recommended (see Figure)	6

For peer review only - http://bmjopen.bmj.com/site/about/guidelines.xhtml

Sample size	14	Estimated number of participants needed to achieve study objectives and how it was determined, including clinical and statistical assumptions supporting any sample size calculations	11
Recruitment	15	Strategies for achieving adequate participant enrolment to reach target sample size	6
**Methods: Assignment of interventions (for controlled trials)**			
Allocation:			
Sequence generation	16a	Method of generating the allocation sequence (eg, computer-generated random numbers), and list of any factors for stratification. To reduce predictability of a random sequence, details of any planned restriction (eg, blocking) should be provided in a separate document that is unavailable to those who enrol participants or assign interventions	NA
Allocation concealment mechanism	16b	Mechanism of implementing the allocation sequence (eg, central telephone; sequentially numbered, opaque, sealed envelopes), describing any steps to conceal the sequence until interventions are assigned	NA
Implementation	16c	Who will generate the allocation sequence, who will enrol participants, and who will assign participants to interventions	NA
Blinding (masking)	17a	Who will be blinded after assignment to interventions (eg, trial participants, care providers, outcome assessors, data analysts), and how	NA
	17b	If blinded, circumstances under which unblinding is permissible, and procedure for revealing a participant's allocated intervention during the trial	NA
**Methods: Data collection, management, and analysis**			
Data collection methods	18a	Plans for assessment and collection of outcome, baseline, and other trial data, including any related processes to promote data quality (eg, duplicate measurements, training of assessors) and a description of study instruments (eg, questionnaires, laboratory tests) along with their reliability and validity, if known. Reference to where data collection forms can be found, if not in the protocol	7, Figure 1,
	18b	Plans to promote participant retention and complete follow-up, including list of any outcome data to be collected for participants who discontinue or deviate from intervention protocols	NA

Data management	19	Plans for data entry, coding, security, and storage, including any related processes to promote data quality (eg, double data entry; range checks for data values). Reference to where details of data management procedures can be found, if not in the protocol	9
Statistical methods	20a	Statistical methods for analysing primary and secondary outcomes. Reference to where other details of the statistical analysis plan can be found, if not in the protocol	9
	20b	Methods for any additional analyses (eg, subgroup and adjusted analyses)	9
	20c	Definition of analysis population relating to protocol non-adherence (eg, as randomised analysis), and any statistical methods to handle missing data (eg, multiple imputation)	NA
**Methods: Monitoring**			
Data monitoring	21a	Composition of data monitoring committee (DMC); summary of its role and reporting structure; statement of whether it is independent from the sponsor and competing interests; and reference to where further details about its charter can be found, if not in the protocol. Alternatively, an explanation of why a DMC is not needed	9
	21b	Description of any interim analyses and stopping guidelines, including who will have access to these interim results and make the final decision to terminate the trial	NA
Harms	22	Plans for collecting, assessing, reporting, and managing solicited and spontaneously reported adverse events and other unintended effects of trial interventions or trial conduct	NA
Auditing	23	Frequency and procedures for auditing trial conduct, if any, and whether the process will be independent from investigators and the sponsor	NA
**Ethics and dissemination**			
Research ethics approval	24	Plans for seeking research ethics committee/institutional review board (REC/IRB) approval	16
Protocol amendments	25	Plans for communicating important protocol modifications (eg, changes to eligibility criteria, outcomes, analyses) to relevant parties (eg, investigators, REC/IRBs, trial participants, trial registries, journals, regulators)	_______________

Consent or assent	26a	Who will obtain informed consent or assent from potential trial participants or authorised surrogates, and how (see Item 32)	7
	26b	Additional consent provisions for collection and use of participant data and biological specimens in ancillary studies, if applicable	NA
Confidentiality	27	How personal information about potential and enrolled participants will be collected, shared, and maintained in order to protect confidentiality before, during, and after the trial	8
Declaration of interests	28	Financial and other competing interests for principal investigators for the overall trial and each study site	18
Access to data	29	Statement of who will have access to the final trial dataset, and disclosure of contractual agreements that limit such access for investigators	18
Ancillary and post-trial care	30	Provisions, if any, for ancillary and post-trial care, and for compensation to those who suffer harm from trial participation	NA
Dissemination policy	31a	Plans for investigators and sponsor to communicate trial results to participants, healthcare professionals, the public, and other relevant groups (eg, via publication, reporting in results databases, or other data sharing arrangements), including any publication restrictions	13
	31b	Authorship eligibility guidelines and any intended use of professional writers	13
	31c	Plans, if any, for granting public access to the full protocol, participant-level dataset, and statistical code	13
**Appendices**			
Informed consent materials	32	Model consent form and other related documentation given to participants and authorised surrogates	Appendix 1
Biological specimens	33	Plans for collection, laboratory evaluation, and storage of biological specimens for genetic or molecular analysis in the current trial and for future use in ancillary studies, if applicable	NA

*It is strongly recommended that this checklist be read in conjunction with the SPIRIT 2013 Explanation & Elaboration for important clarification on the items. Amendments to the protocol should be tracked and dated. The SPIRIT checklist is copyrighted by the SPIRIT Group under the Creative Commons "Attribution-NonCommercial-NoDerivs 3.0 Unported" license.

For peer review only - http://bmjopen.bmj.com/site/about/guidelines.xhtml
